# From blur to blueprint: a fuzzy delphi methodology for evaluating early-stage emerging technologies—the case of end-of-life automotive traction battery disassembly

**DOI:** 10.1038/s41598-026-50592-1

**Published:** 2026-05-19

**Authors:** Max Rettenmeier, Mauritz Möller, Alexander Sauer

**Affiliations:** 1 Graduate School of Excellence advanced Manufacturing Engineering (GSaME), University of Stuttgart, Nobelstraße 12, 70569 Stuttgart, Germany; 2 TRUMPF Laser- und Systemtechnik SE, Johann-Maus-Straße 2, 71254 Ditzingen, Germany; 3 Fraunhofer-Institute for Manufacturing Engineering and Automation (IPA), Nobelstraße 12, 70569 Stuttgart, Germany; 4 Institute for Energy Efficiency in Production (EEP), University of Stuttgart, Nobelstraße 12, 70569 Stuttgart, Germany

**Keywords:** Technology evaluation, Automotive battery, End-of-life battery, Disassembly, Dismantling, Fuzzy delphi, Energy science and technology, Engineering, Environmental sciences

## Abstract

Recycling end-of-life automotive traction batteries is paramount in reducing greenhouse gas emissions and mitigating supply chain dependencies associated with critical raw materials utilized in producing electric vehicle batteries. Deploying disassembly technologies represents a crucial step in the pretreatment process, enhancing the recycling process’s sustainability and profitability. This study evaluates disassembly technologies based on a multi-stage expert-driven research approach. We present a systematic stakeholder identification and analysis for the disassembly process step, resulting in the 14 relevant stakeholders. 65 expert interviews were then conducted with the relevant stakeholders and yielded a corpus of 16 evaluation criteria. Each criterion underwent a delphi evaluation process with 13 experts to ascertain its relative importance. Ultimately, another delphi panel was utilized to evaluate each disassembly technology conjunction with all identified criteria. To address potential uncertainties, the fuzzy delphi method was employed, resulting in two rounds until a consensus was achieved. Laser cutting and shredding emerged as particularly promising technological approaches, recommended for consideration by industrial stakeholders in the expansion of their facilities and by policymakers in the formulation of their regulatory environment. The developed methodological approach aims to systematically evaluate early-stage emerging technologies and has been validated by the given use case.

**Supplementary Information:**

The online version contains supplementary material available at 10.1038/s41598-026-50592-1.

## Introduction

The Paris Climate Agreement marks a pivotal milestone in the global endeavor to tackle the mounting threats posed by climate change^1^. However, the agreed objectives are currently at risk, given that global efforts to reduce greenhouse gas (GHG) emissions are insufficient^2^. Private cars and vans accounted for over 25% of global oil consumption and approximately 10% of global energy-related CO_2_ emissions in 2022^3^. Consequently, the acceleration of electric mobility represents a paramount industrial imperative of the 2020s and 2030s to decarbonize the transportation sector. One of the challenges associated with the transition to electrified powertrains is the availability and sustainability of battery packs^4,5^. The automotive electric vehicle lithium-ion traction battery pack (EVB) contains several materials with limited global availability, creating a vulnerable supply chain for regions with low natural resource endowments in this field^6^. In light of this, the European Union has designated certain materials as critical or strategic respectively^7^. Critical raw materials (CRMs) must demonstrate a certain level of supply risk and economic importance. In contrast, strategic raw materials (SRMs) are crucial for technologies that facilitate the dual transition to a green and digital economy, along with defense and aerospace objectives^7^. The list of these materials includes many substances indispensable for EVB production^7^:

- Aluminum (cathode current collector of the battery cell - CRM).
- Copper (anode current collector of the battery cell - SRM).
- Cobalt (cathode active material - CRM & SRM).
- Lithium (cathode active material - CRM & SRM).
- Nickel (cathode active material - SRM).
- Manganese (cathode active material - CRM & SRM).
- (Natural) Graphite (anode active material - CRM & SRM).

Furthermore, the EVB accounts for 40–60% of the GHG emissions associated with producing an electric vehicle (EV)^8^. From this, mining and refining contribute 26%, and active material production accounts for 32% of the GHG emissions from battery production^8^. The EVB and its materials are thus not only critical in terms of supply chain security but also in terms of GHG emissions. One strategy for diminishing the GHG emissions of these materials and enhancing supply chain resilience in regions with limited natural resources of battery materials is the incorporation of recycled materials^9,10^.

It is, therefore, essential in the pursuit of sustainability and accelerated adoption of electric mobility to facilitate the recycling of retired EVBs^11,12^. The end-of-life (EOL) stage of an EVB is defined as the point at which the device’s remaining capacity reaches 80%, as indicated by the state of health^13^. Upon reaching the EOL stage, EVBs are eligible for various Re-X routes, including remanufacturing, reuse, or recycling^14^. Even if EOL EVBs are remanufactured or reused, this merely postpones the point at which they are recycled. Hence, our study focuses on the recycling of EOL EVBs.

**Fig. 1:**
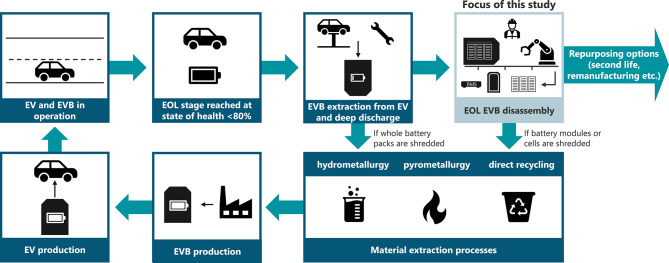
EVB recycling process (according to Rettenmeier et al.^21^).

For an overview of the process steps for EVB recycling, we refer to existing studies, for example, by Baum et al.^15^, Ciez et al.^16^, Gaines^17,18^, Harper et al.^14^, Sommerville et al.^19^ and Velázquez-Martínez et al.^20^. To provide a simplified summary, Fig. 1 illustrates the battery recycling process.

**Fig. 2:**
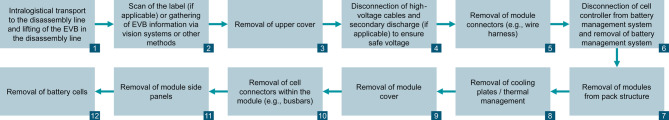
EOL EVB disassembly process according to Rettenmeier et al.^21^.

This study focuses on the disassembly as a pretreatment step in the recycling of EOL EVBs^22^. Figure 2 illustrates the disassembly process for EOL EVBs according to Rettenmeier et al.^21^. The disassembly process considerably diminishes the impurity of the materials entering subsequent recycling stages, aimed at separating valuable materials^23^. Furthermore, disassembly minimizes the mass flow of materials for downstream processing, thereby enabling either the downsizing of machinery or an increase in purer mass flows^24,25^. As the terms disassembly and dismantling are used interchangeably in the literature, we will also employ these terms synonymously.

Although the disassembly step is of great importance to the overall EVB recycling process, numerous challenges have been identified in this area, as outlined in the studies by Rettenmeier et al.^21,26^. Regarding the product, the EOL EVB, a high degree of variety in combination with non-detachable joints and restricted component accessibility present significant challenges for the disassembly process^21^. In operation, manual processing, lack of a skilled workforce, and lack of automated workflows present significant challenges^21,27^. Furthermore, the product’s condition as an EOL item introduces uncertainty to the disassembly process^21^. This uncertainty can also be related to the safety condition of the EOL EVB, which must be handled in the disassembly process. In light of these considerations, it becomes evident that the disassembly of the EOL EVB is a highly complex endeavor, comprising numerous challenges that must be addressed through technological solutions. It is, therefore, essential to conduct a comprehensive and holistic evaluation of these technologies to inform decision-making processes and ensure the development of an efficient and sustainable disassembly process in the future.

In a recent study, Rettenmeier et al.^21^ investigated the process of EOL EVB disassembly to identify and categorize different disassembly technologies. Another study yielded a preliminary compact benchmark analysis of EOL EVB disassembly technologies^28^. Building upon the recent research in the field, this study aims to evaluate and benchmark these technologies for the disassembly of structural components of EOL EVBs on pack level to provide industrial decision makers with guidance for investment planning. Furthermore, we aim to inform policy makers about the benefits and drawbacks of the different technological derivatives to facilitate the development of appropriate policy and guide future subsidies in this field. Ultimately, the methodology we develop and apply can be used as a general framework to evaluate early-stage emerging technologies, as this is a major challenge^29^.

The article is structured as follows. The “State of the art” section provides a state-of-the-art overview, followed by elaborating our methodological setup in the “Methods” section. The results are presented and discussed in the “Results and discussion” section before the study concludes with the “Conclusions” section.

## State of the art

The disassembly of EOL EVBs attracts an increasing interest from the scientific community. However, many publications address the disassembly step as a subtopic within the context of battery recycling. Consequently, a substantial corpus of general and superficial information on the disassembly of EOL EVBs pervades the literature. In-depth investigations on disassembly technologies and their respective separation functionalities are scarce and often narrowly focused on specific, detailed sub-aspects, such as digital technologies. Furthermore, a significant number of studies concentrate on a single disassembly technology or even a specific aspect of it. Thus, conducting a comprehensive and systematic evaluation of disassembly technologies for informed decision-making in industrial contexts is challenging. Table 1 demonstrates how our study addresses this challenge by encompassing all relevant disassembly technologies with a systematic benchmark, enhancing the existing scientific literature in this field. Given the rapid evolution of the battery recycling and disassembly industry, we additionally provide a robust methodology that can be repeatedly conducted in light of technological changes and advancements.

As Table 1 illustrates, an examination of the existing literature reveals that numerous articles address EOL EVB disassembly from a variety of perspectives. However, fewer studies concentrate on non-destructive and semi-destructive disassembly technologies, providing insights into multiple disassembly techniques. Definitions for non-destructive and semi-destructive disassembly technologies can be found in the study by Rettenmeier et al.^21^. In addition to the literature provided in Table 1, the publication from Hathaway et al.^30^ should be mentioned as a perspective focused on robotics. It is essential to consider a range of disassembly technologies to provide a comprehensive benchmark. Many studies concentrate on a single or pair of disassembly technologies, most commonly milling or unscrewing. For instance, Choux et al.^31^, Kay et al.^32^, and Meng et al.^33^ consider multiple disassembly technologies, although only a select few are examined in comparison to Rettenmeier et al.^21^. The authors then briefly discuss the selected disassembly technologies, which can be regarded as a starting point for establishing a benchmark. On this basis, a preliminary benchmark was conducted by Rettenmeier et al.^28^. In this study, however, they rely exclusively on the evaluation criteria identified by Herrmann et al.^34^ and the disassembly technologies identified by Fleischer et al.^35^, equipped with evaluations based on the current literature. As this was only intended as a preliminary starting point by the authors, we now conduct a full-scale benchmarking study with the entire range of technologies subsequently identified in the study by Rettenmeier et al.^21^. To identify and weigh relevant evaluation criteria, we now draw not only on evidence-based research but also on a qualitative, expert-based methodology. As a result, we extend the current state of research in terms of scope and depth and from a methodological point of view.

**Table 1:** Overview of the relevant literature for EOL EVB disassembly (√ = topic covered in the according article | (√) = topic partly covered with limitations in the according article).

Article	EOL EVB disassembly focus	Consideration of destructive, non-destructive, semi-destructive disassembly technologies	Consideration of multiple disassembly technologies	Benchmark of disassembly technologies
Fervorari & Colledani^36^	√			
Böttcher & Rettenmeier^37^	√			
Gerlitz et al.^38^	√			
Glöser-Chahoud et al.^23^	√			
Hellmuth et al.^39^	√			
Hertel et al.^40^	√			
Kaarlela et al.^41^	√			
Klohs et al.^42^	√			
Li et al.^43^	√			
Wu et al.^44^	√			
Alfaro-Algaba & Ramirez^45^	√	√		
Choux et al.^31^	√	√		
Miloradović et al.^46^	√	√		
Rettenmeier et al.^47^	√	√		
Baazouzi et al.^48^	√	√	√	
Beghi et al.^49^	√	√	√	
Rettenmeier et al.^21^	√	√	√	
Rosenberg et al.^50^	√	√	√	
Wu et al.^44^	√	√	√	
Choux et al.^31^	√	√	√	(√)
Bachler & Rettenmeier^51^	√	√	√	(√)
Kay et al.^32^	√	√	√	(√)
Meng et al.^33^	√	√	√	(√)
Rettenmeier et al.^28^	√	(√)	(√)	(√)
This study	√	√	√	√

Given the methodological focus of our article on the evaluation of early-stage emerging technologies, we also present the state of the art in this domain. As illustrated in Table 2, several studies deal with the field of technology evaluation. However, when evaluating early-stage emerging technologies, the existing research field becomes considerably narrower due to the high uncertainty and smaller data foundation for the evaluation process. As the existing literature demonstrates, there are studies detailing fuzzy delphi approaches for evaluating technologies, including those in the field of early-stage emerging technologies. In contrast to extant studies, we create a multi-perspective benchmark without limiting the evaluation field to, for example, economic or ecological criteria. Furthermore, we augment existing fuzzy delphi approaches for early-stage emerging technology evaluation, as exemplified by Faber et al.^52^ and Huang et al.^53^, by offering a granular methodology that encompasses systematic stakeholder identification, criteria identification, criteria consolidation, criteria weighting, and technology evaluation. A comparison of our methodology with existing research reveals that it adopts a more systematic approach, particularly in the initial stages of stakeholder identification and criteria identification, compared to the approaches employed by these studies. These preliminary stages of the methodology are paramount, as they lay the foundation for the subsequent course of the evaluation. To address this gap, we have formulated a systematic procedure encompassing stakeholder and criteria identification, combined with fuzzy delphi weighting of criteria and evaluation of technologies, which serves as a blueprint for the research community. In addition, our study is novel regarding the field of application, which is dedicated to EOL EVB disassembly.

**Table 2:** Overview of the relevant literature for technology evaluation.

Article(s)	Method	Evaluated categories	Technology maturity	Thematic and technological focus
Bergerson et al.^54^; Frischknecht et al.^55^; Hung et al.^56^; Moni et al.^57^; Thonemann et al.^58^	LCA	Environmental	Emerging / early-stage	No specific focus
Chang et al.^59,60^	Fuzzy-delphi	Economic, environmental, technological, societal	Established	Hydrogen production technologies
Cho & Lee^61^	Fuzzy-delphi	Economic/commercial, technological	Established	No specific focus
Faber et al.^52^	LCA, TEA	Economic, environmental, technological	Emerging / early-stage	Carbon Management Technologies
Haag et al.^62^; Schot & Rip^63^; Saffie et al.^64^	Overview	Overview	No specific focus	No specific focus
Hsu et al.^65^	Fuzzy-delphi	Economic, environmental, technological	Established	Lubricant regenerative technology selection
Hsu & Chang^66^	Fuzzy-delphi	Economic, environmental, technological, societal	Established	Hydrogen production technologies
Huang et al.^53^	Fuzzy-delphi	economic, social, technological	emerging / early-stage	No specific focus
Ji-wu et al.^67^	Nominal group analysis, analysis network process, stakeholder analysis, bibliometric analysis, patent analysis	economic/commercial	emerging / early-stage	No specific focus
Lee & Chou^68^	Fuzzy-delphi	economic, technological	emerging	3DIC Integration Technologies
Martinez et al.^69^	LCA	Environmental	Emerging / early-stage	Emerging hydrogen technologies
Schuh et al.^70^	General methodology by Ulrich ^71^	Application-based utility potential	Emerging / early-stage	Manufacturing technologies
This study	Fuzzy-delphi combined with expert interviews	Economic, environmental, technological	Emerging / early-stage	Disassembly technologies for EOL EVBs

Although there is an expanding corpus of literature addressing EOL EVB disassembly and technology evaluation, a notable research gap persists. The extant literature on the subject either concentrates on individual disassembly technologies or is lacking a comprehensive and systematic benchmarking approach across multiple technological alternatives. Concurrently, methodological contributions to the evaluation of emerging technologies are frequently constrained in scope, particularly with regard to the integration of structured stakeholders and the development of criteria under conditions of high uncertainty. Consequently, there is a notable absence of robust, transparent, and reproducible approaches that enable decision-makers to systematically assess emerging technologies based on a multi-dimensional techno-economic perspective.

In light of these considerations, the objective of this study is to develop and validate a comprehensive methodological framework for the evaluation of early-stage emerging technologies. The present study aims to address the following research question: How can early-stage emerging technologies be systematically assessed based on techno-economic criteria, using the example of EOL EVB disassembly technologies?

This research makes two methodological contributions to technology management literature. First, it combines stakeholder-driven criteria identification, enhanced fuzzy delphi weighting, and a structured benchmarking approach. Second, it provides an empirically grounded application in the domain of EOL EVB disassembly.

## Methods

### Methodological framework

**Fig. 3:**
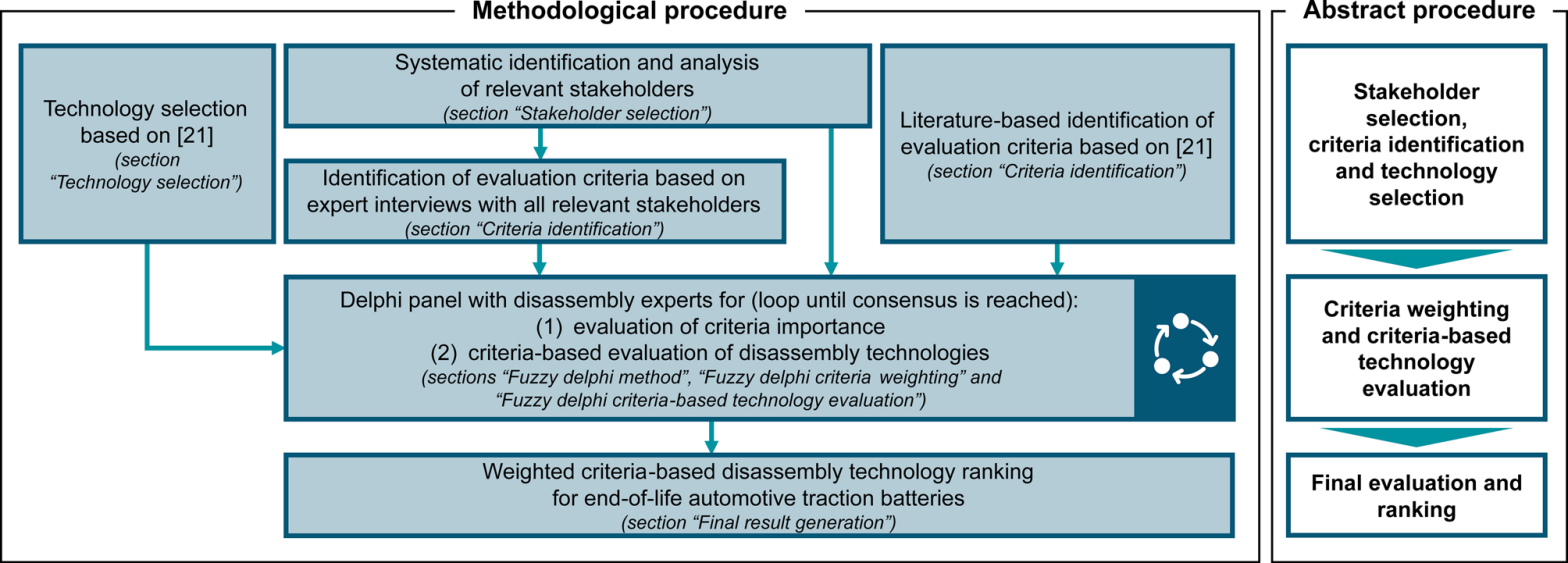
Methodological framework for this study.

To evaluate and benchmark disassembly technologies for EOL EVBs, we employ the methodological framework illustrated in Fig. 3. A comprehensive delineation of the methodological details is provided in the following sections. Within the scope of this paper, several interviews and delphi panel rounds were conducted. Regarding those, informed consent was obtained from all individual participants included in the study. The participants have also consented to the submission of the case report to the journal.

The methodological procedure follows a structured, multi-stage approach to ensure a systematic and robust evaluation of disassembly technologies. Initially, the relevant technologies are identified through a comprehensive examination of existing literature, thereby establishing a consistent technological scope (see “Technology selection” section). In parallel, a comprehensive stakeholder analysis is conducted to identify all relevant stakeholders along the value chain, forming the basis for subsequent expert involvement (see “Stakeholder selection” section). The following evaluation criteria are derived from the results of qualitative expert interviews with the relevant stakeholders (see “Criteria identification” section) and complemented by a literature-based analysis to ensure comprehensiveness and theoretical foundation (see“Criteria identification” section). The consolidated set of criteria is subsequently subjected to a fuzzy delphi process involving a panel of disassembly experts. In the course of iterative rounds, experts evaluate the significance of the criteria and the performance of the selected technologies against these criteria until a consensus is achieved (see sections “Fuzzy delphi method”, “Fuzzy delphi criteria weighting” and “Fuzzy delphi criteria-based technology evaluation”). Ultimately, the resulting weighted criteria are applied to generate a comparative ranking of disassembly technologies for end-of-life automotive traction batteries (see “Final result generation” section).

### Technology selection

This study is based on the following disassembly technologies:

- Crushing (wet / dry).
- Knife / shear cutting.
- Laser cutting.
- Milling.
- Rotary cutting tools (incl. saw and angle grinder.
- Water-jet cutting.
- Unscrewing.
- Toolless manual removal or separation operations.
- Excluded:
  - Desoldering.
  - Drilling.
  - Oxy fuel cutting.
  - Plasma cutting.

Further information and references on the selection of those disassembly technologies are reported in Table A.1 and are consolidated below.

The crushing of EOL EVBs is deemed a disassembly technology with a destructive character. In some contexts, articles differentiate between shredding and disassembling, thus not categorizing shredding as a disassembly technology^31,72^. However, given the competitive nature of these technologies, it is essential to also consider the shredding procedure as a benchmark for disassembly technologies. Consequently, our study includes shredding technology, classifying it as a destructive disassembly technology. We always compared shredding complete EVBs on pack level with the other disassembly approaches. As desoldering could not be identified as a researched or used technique in this field with the exception of Zhao^73^, this technology was excluded from consideration in order to limit the number of technologies and, consequently, the evaluation efforts. Furthermore, given the absence of concrete real-life illustrations, with the exception of the investigations conducted by Zhao^73^, it was assumed to be challenging for the experts to evaluate the role of the technology, due to the lack of data, examples and experiences. However, should the technology be applied more widely in future, it is possible that it will be included in the scope of subsequent investigations.

As illustrated in Fig. 2, a significant proportion of the disassembly process involves structural components, encompassing the non-destructive disassembly of screw connections and the semi-destructive disassembly of steel or aluminum components. In this context, unscrewing or cutting processes are widely accepted for the separation of those components.

Furthermore, drilling was excluded from consideration due to its comparable characteristics with milling. From a technical standpoint, milling can be viewed as a broader concept that encompasses drilling, particularly when axial tool movement is employed. It can be concluded that, in principle, a drilling operation can be replicated using milling equipment with appropriate tool paths and feed strategies. This is particularly important in the context of EOL EVB disassembly, where the flexibility of tool trajectories is often necessary due to the variability of components. The inclusion of both milling and drilling would therefore introduce redundancy without providing additional insights into process capabilities. Consequently, the incorporation of milling adequately embodies the functional dimensions of drilling within this specific application domain.

Additionally, its utilization is restricted to specific connections, such as stuck screws, where milling could be equally applied. In addition, oxyfuel and plasma cutting were excluded from consideration because these disassembly technologies were merely mentioned in the study by Fleischer et al.^35^. In this study, Fleischer et al.^35^ did not conduct any dedicated investigations into these technologies, only a brief mention was made. As no further examinations of these two approaches were identified, they were excluded from the study. Even though water-jet cutting was only referenced in the work of Fleischer et al.^35^, according to Rettenmeier et al.^21^, this technology was incorporated into our analysis. This decision was motivated by numerous patents that address water-jet as a method for disassembling EOL EVBs^74,75^.

### Stakeholder selection

As a precursor to the expert-based methodological steps, we have identified the relevant stakeholder groups within the field of technology evaluation for EVB disassembly.

**Fig. 4:**
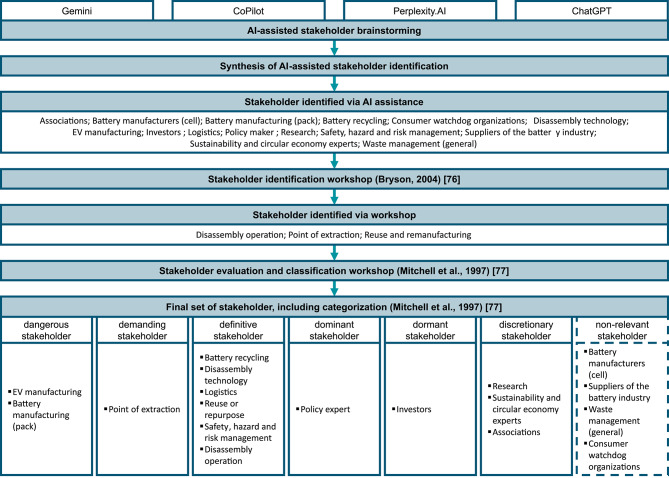
Procedure to identify relevant stakeholders.

To identify appropriate stakeholders, we employed the framework illustrated in Fig. 4. We conducted an artificial intelligence (AI) assisted approach as the initial basis for an established workshop format, according to Bryson^76^. The AI-assisted stakeholder identification started with inserting the same search prompt in four popular state-of-the-art generative AI tools (see Appendix B). It is important to mention that the AI tools were used exclusively used to identify an exploratory set of stakeholders.

The results of the AI tools were then discussed, filtered, and extended in a systematic procedure. For this reason, we do not solely rely on the results given by the AI tools, but rather use them as an initial source of inspiration. The results from the different tools are reported in Tables B.1 and B.2. We then consolidated the results from the AI tools and removed redundancies, which is granularly reported in Table B.3. Those initial stakeholder groups formed the basis for the workshop to identify further stakeholders. Hereby, the stakeholders identified by AI provided a solid base and gave fruitful inspiration, which is why only a few further stakeholders were identified during the workshop as shown in Fig. 4. We also adjusted the naming of some stakeholders, as shown in Table B.4. We then conducted a further workshop according to the methodology presented by Mitchell et al.^77^. The stakeholders were categorized according to Mitchell et al.^77^ by rating them in the categories of power, urgency, and legitimacy (see Table B.4). The stakeholders that were determined to be non-relevant were excluded from the scope of our considerations. The remaining stakeholder groups form the basis for the expert selection in the subsequent “Criteria identification” section.

### Criteria identification

The starting point for the evaluation of disassembly technologies is identifying the relevant criteria. The identification process was conducted through expert interviews with all relevant stakeholders identified in the previous “Stakeholder selection” section. We conducted 65 expert interviews across the battery recycling industry (see Table C.1). We selected the experts according to their current job position, which must be in relation to EVB disassembly technologies in at least one of our identified stakeholder roles. Years of experience in the EVB disassembly field were not taken as decisive factors since EOL EVB disassembly is a very young industrial field. Until now, many disassembly processes and technologies have not been established industrially in this field, which is why we do not require a certain number of years of experience in this field. Furthermore, EVB disassembly technologies are usually adapted from other applications and industries with similar requirements, so professional experience in general is more meaningful here than working experience in the EVB disassembly field. We further gathered the worker type of the experts to show that we acquire data across all hierarchy levels in an organization, leveraging the variety of opinions. All details from the experts, including experience level, worker type, and stakeholder group(s), can be found in Table C.1. Regarding the stakeholder group, not only the current position of the experts was taken into account, but also their professional background was considered. As policy makers, we also took into consideration public institutions, which exert a significant influence on policy processes.

The brief interview encompassed a single question (“What criteria would you use to evaluate disassembly technologies for EOL EVBs?”), which addressed the objective of identifying pertinent criteria for evaluating disassembly technologies for EOL EVBs. The interviews yielded a total of 16 distinct criteria. An exhaustive delineation of the results obtained from the criteria identification process is presented in the “Evaluation criteria” section.

Along with the expert-based approach in which we empirically identify relevant evaluation criteria, we enrich those investigations with references from the literature. We, therefore, rely on the systematic literature analysis we have already published. The search documentation and methodology are documented in Rettenmeier et al.^21^. This further strengthens the validity of our criteria set since we take them not only from existing research but also from a broad base of experts, systematically selected with the explained stakeholder approach (see “Stakeholder selection” section).

### Fuzzy delphi method

The delphi method was employed in this study as a leading approach to achieve consensus among an expert panel. The delphi method was initially developed by the RAND Corporation to generate consensus in the context of forecasting. It gained public interest after Dalkey and Halmer^78^ published the principles of the experimental delphi application. Following its initial implementation in the sphere of military strategy, the delphi method was rapidly adopted by several other domains^79^. Nowadays, the delphi expert method is one of the most prominent qualitative research methods in decision-making across a range of scientific domains, including social, medical and health sciences.

Building on this background, researchers have also applied the methodology to technological forecasting, as evidenced by the work of English and Kernan^80^. Subsequently, the delphi method has also gained recognition as a reliable approach for decision-making in various subdomains of technology management, as evidenced by the existing literature^81–84^(see further examples in Table 2).

The field of EOL EVB disassembly is currently undergoing an industrial ramp-up phase, during which emerging technologies are being developed. Moreover, the field of EOL EVB disassembly is comparatively novel and early-stage, given the recent advent of electric mobility as a technology, in comparison to, for instance, combustion-based mobility concepts. This necessitates the use of an expert-based, qualitative research approach, as the requisite deep technological information essential for technology evaluation is not widely accessible to the general public but is instead limited to specialized stakeholders and individuals active in this domain. Other research methods, such as a quantitative survey, would be exceedingly challenging given the limited number of potential respondents, making gathering sufficient responses for a quantitative study unfeasible. Given the established nature of the delphi method, its extensive use across numerous scientific domains, and its dedicated application in technological realms, we thus elected to employ the expert-based delphi panel in our study.

To further enhance the delphi-based approach, we expanded our methodological approach by integrating the principles of fuzzy logic. Since Zadeh^85^ invented fuzzy logic as a mathematical method to characterize states between zero and one, offering an alternative to the classical Boolean approach, it provides the benefit that uncertainties can be mathematically handled^86^. This is particularly vital given that technology evaluation in an early-stage emerging field is concerned. The integration of fuzzy logic into delphi methods has been extensively documented in scientific literature, as exemplified in the “State of the art” section.

A delphi panel was utilized to determine the criteria weighting and the criteria-based technology evaluation. This panel comprised 13 experts specializing in disassembly technologies. Despite the delphi expert panel consisting of 13 participants, this panel size aligns with established methodological standards and is commonly found in similar fuzzy delphi studies, particularly those with a qualitative or exploratory focus (Hsu et al.^65^: 9 experts^59^; 17 experts). Moreover, Linstone^87^ suggests a panel size of at least seven experts, while Ameyaw et al.^88^ investigated a majority of studies employing 8–20 experts. The use of seven experts is also employed by Dalkey and Helmer^78^, which is one of the earliest publications with the delphi method from the RAND Corporation. The aforementioned numerical values are consistent with the recommendation of a panel size comprising 10–15 individuals to ensure reasonable outcomes, as substantiated by Ziglio^89^.

The size of our panel, which consists of 13 experts, is consistent with this range and the numbers from the given literature. In the context of the delphi method, representativeness is not achieved through statistical sampling but rather through the informed evaluation of individuals who hold specialized knowledge and insights relevant to the topic under investigation (see selection in Table 3). The manageable panel size also facilitated consistent participation across all delphi rounds, eliminating any dropout and ensuring continuity in the development of consensus, which is central for delphi studies^90^.

In contrast to the experts involved in the initial criteria identification, the selection process focused exclusively on individuals whose professional practice involved the disassembly of EOL EVBs. However, the composition of the aforementioned panel of experts reflects a diverse range of disciplines relevant to the field under consideration, including research, machine building, recycling, dismantling, and OEMs (Original Equipment Manufacturers). We thereby demonstrate the simultaneous incorporation of academic and industrial perspectives.

Furthermore, the inclusion of diverse perspectives along the disassembly supply chain is ensured, encompassing machine builders and operating companies, such as recyclers and dismantlers, to achieve comprehensive representation. Moreover, the producers of the battery pack architectures are also included as OEMs. Of further note is the fact that all of the aforementioned experts are employed by different companies or institutions, thus ensuring a wide range of perspectives within the panel. All experts from the panel were also involved in the initial criteria identification process (see “Criteria identification” section). However, the two interviews and ratings were conducted separately. An overview of the experts and the characteristics of the delphi panel is reported in Table 3. As previously described for the expert selection during the criteria identification, we did not require specific years of experience in the domain, given that EOL EVB disassembly is a relatively new field. In light of the rapid developments in the field of EOL EVB disassembly, it was deemed essential to administer the two delphi rounds within a relatively narrow time frame. Notably, the delphi panels utilized for the criteria weighting and criteria-based technology evaluation were identical.

To enhance transparency regarding the depth of expertise and potential sources of bias within the expert panel, additional information on participant selection and qualification criteria is provided. All experts included in the study were required to be actively engaged in topics related to battery disassembly at the time the study was conducted. This approach ensures that their assessments are firmly rooted in up-to-date industrial or research practices. Beyond the scope of mere involvement, particular emphasis was placed on ensuring that all experts possessed a broad and comparative understanding of multiple disassembly technologies. Within the fuzzy delphi framework, this requirement was considered essential for a balanced evaluation, as the methodology relies on informed judgments across a range of technological alternatives. In accordance with this objective, a group of experts with a wide range of professional backgrounds, including industry and academia, and who were involved in different stages of the battery disassembly chain, were selected for participation in the study. This diversity in expertise and perspective was intended to reduce the probability of systematic bias toward any particular technology and to support a more robust and balanced consensus formation process.

**Table 3:** Characteristics of the delphi panel.

Expert ID	Industry	Job title	Years of experience	Date round 1 (YYYY-MM-DD)	Date round 2 (YYYY-MM-DD)
Expert 1	Academic	Professor	8+	2024-12-16	2025-01-14
Expert 2	Machine builder	CEO	27+	2024-12-16	2025-01-15
Expert 3	Recycler/dismantler	Senior expert	15+	2024-12-17	2025-01-10
Expert 4	Academic	Research associate	5+	2024-12-17	2025-01-14
Expert 5	Recycler/dismantler	Senior expert	9+	2024-12-18	2025-01-13
Expert 6	Oem	Senior expert	21+	2024-12-18	2025-01-10
Expert 7	Machine builder	Senior expert	9+	2024-12-18	2025-01-10
Expert 8	Machine builder	Senior expert	7+	2024-12-18	2025-01-10
Expert 9	Recycler/dismantler	Expert	4+	2024-12-18	2025-01-09
Expert 10	Machine builder	Expert	2+	2024-12-19	2025-01-15
Expert 11	Academic	Research associate	5+	2024-12-19	2025-01-23
Expert 12	Recycler/dismantler	CEO	20+	2024-12-20	2025-01-23
Expert 13	Recycler/dismantler	Senior expert	12+	2024-12-20	2025-01-08

In order to preserve the anonymity of the experts, each of them was interviewed separately. According to Murry and Hammons^91^, Linstone and Turoff^92^, and Okoli and Pawlowski^93^, anonymity is one of the central pillars of delphi methods. Anonymity preserves the ability of individuals to form their own opinions independently of influences. The technical details of the delphi panel process are delineated in the “Fuzzy delphi criteria weighting” and “Fuzzy delphi criteria-based technology evaluation” sections. From an organizational perspective, the experts were interviewed individually, and their identity was kept anonymous from the other experts. As illustrated in Fig. 3, the experts evaluated the criteria concerning their importance and the technologies in a criteria-oriented manner regarding their performance.

### Fuzzy delphi criteria weighting

Once the pertinent criteria had been identified in accordance with the procedures prescribed in the “Criteria identification” section, a weighting of the criteria was conducted through a delphi panel. This process involved the evaluation of the importance of each criterion by the experts, utilizing a seven-point evaluation scale (see Table 4). Lewis^94^ investigated the correlation between a seven-point scale and a five-point scale and concluded that the seven-point scale offers a higher correlation with the observed significance level. Furthermore, Miller^95^ postulates that the human mind is capable of assessing within a range of seven categories, which substantiates the rationale for employing a seven-point evaluation scale as the optimal range. Additionally, we explored the feasibility of utilizing a six-point scale without the neutral option. However, in light of Simms et al.^96^ findings that the difference between these scales is minimal to non-existent, we ultimately opted to include the neutral option. Therefore, a seven-point evaluation scale was employed for weighting the criteria, as illustrated in Table 4.

**Table 4:** Evaluation scale for criteria weighting.

Evaluation scale (linguistic variables)	Fuzzy numbers			Center of gravity
	$\:{f}_{Ec1}$	$\:{f}_{Ec2}$	$\:{f}_{Ec3}$	
Not important at all	0.0	0.0	0.1	0.0333
Mostly not important	0.0	0.1	0.3	0.1333
Somewhat not important	0.1	0.3	0.5	0.3000
Neither important nor unimportant	0.3	0.5	0.7	0.5000
Somewhat important	0.5	0.7	0.9	0.7000
Mostly important	0.7	0.9	1.0	0.8667
Extremely important	0.9	1.0	1.0	0.9667

After the experts evaluated each criterion with the linguistic variables from Table 4, the ratings were associated with the fuzzy numbers shown in Table 4^97^. We employed the linear triangular fuzzy approach because the triangular fuzzy set has already been used in a large number of other studies in this field and also limits complexity^59,61,64–66,98–100^. As illustrated in Table 4, the triangular fuzzy logic was linked to each linguistic variable, thereby ensuring the transferability of the linguistic variables into numerical outcomes, which are subsequently processed further.

Since we had 13 experts ($\:E\in\:\left[1;13\right]$ as documented in Table 3) in our delphi panel and 16 criteria ($\:c\in\:\left[1;16\right]$ as later documented in Table D.1) for weighting, there were 208 items ($\:{i}_{Ec})$ for each round of evaluation (see Eq. 1). $\:{r}_{n}$ serves as a round index for $\:n$ rounds.

1 $$\mathrm{Number}\, \mathrm{of} \:{i}_{Ec}=E\cdot c$$

We then employed the distance to mean ($\:DtM$) as a metric to filter the ratings from round 1, which must be presented to the experts for reevaluation (see Eq. 2). As a method to determine the distance to mean, we have employed the vertex distance, as shown in several previous studies^60,66,99,101^.

2 $$\:Dt{M}_{{i}_{Ec}\left({r}_{1}\right)}=\sqrt{\frac{1}{3}\left({\left({f}_{Ec{1}_{{r}_{1}}}-\frac{\sum\nolimits_{c=1}^{16}{f}_{Ec{1}_{{r}_{1}}}}{16}\right)}^{2}+{\left({f}_{Ec{2}_{{r}_{1}}}-\frac{\sum\nolimits_{c=1}^{16}{f}_{Ec{2}_{{r}_{1}}}}{16}\right)}^{2}+{\left({f}_{Ec{3}_{{r}_{1}}}-\frac{\sum\nolimits_{c=1}^{16}{f}_{Ec{3}_{{r}_{1}}}}{16}\right)}^{2}\right)}$$

As a threshold, we have set $\:Dt{M}_{{i}_{Ec}\left({r}_{n}\right)}\le\:0.1\stackrel{-}{6}$, as this is the median distance between the center of gravity (CoG) values of the evaluation scale stages (see Table 4). Further elaborations on the methods for calculating the CoG can be found in the “Final result generation” section. The threshold of $\:0.1\stackrel{-}{6}$ was determined based on an analysis of the CoG values derived from the fuzzy evaluation scale employed in this study (see Table 4). The calculated inter-CoG distances ranged from 0.1 to 0.2, with outer categories exhibiting smaller distances (e.g., 0.1 and $\:0.1\stackrel{-}{6}$) and central categories showing larger distances (up to 0.2). The median value of these distances ($\:0.1\stackrel{-}{6}$) was selected as a robust measure of the central tendency of the scale. This threshold implies that expert assessments with deviations from the mean of up to $\:0.1\stackrel{-}{6}$ can still be considered consistent with the prevailing evaluative tendency. However, as responses deviate further, especially when they trend toward the opposite evaluative direction, they surpass the acceptable range. Such significant deviations signal the need for re-examination in a subsequent round of the delphi study. By enforcing this threshold, the methodology ensures that only responses aligning closely with the central consensus are retained. Meanwhile, those exhibiting significant discrepancies undergo additional examination, thereby enhancing the reliability and validity of the consensus-building process. Several publications used a threshold of 0.2^59,60,66,102–111^. A considerable number of the aforementioned publications cite Cheng and Lin^112^ as a primary source for this value. However, even this publication merely defines this value. For this reason, we took the opportunity to logically develop an own threshold value that is stricter ($\:0.1\stackrel{-}{6}<0.2$) as the common value of 0.2.

After filtering all $\:{i}_{Ec}\left({r}_{1}\right)$ according to $\:Dt{M}_{{i}_{Ec}\left({r}_{n}\right)}\le\:0.1\stackrel{-}{6}$, the items $\:{i}_{Ec\_re}\left({r}_{1}\right)$ are left. The remaining items were then presented to the experts. To show the discrepancy between their evaluation and the evaluations from the other experts, we have shown a distribution of how many experts rated the according criteria in the seven evaluation stages. Since experts from different domains have a different corpus of knowledge, additionally the justifications for rating the items in $\:{r}_{1}$ were documented, consolidated and presented to the experts, so that they know why other experts rated the item differently. Ultimately, experts were not forced to change their opinion during the reevaluation process, they could also stay with their opinion from $\:{r}_{1}$. The reevaluated items are named $\:{i}_{Ec\_ev}\left({r}_{1}\right)$.

After the experts reevaluated the items, a new set of items was created as the base for $\:{r}_{2}$ (see Eq. 3).

3 $$\:{i}_{Ec}\left({r}_{2}\right)=({i}_{Ec\_ev}\left({r}_{1}\right)+({i}_{Ec}\left({r}_{1}\right)-{i}_{Ec\_re}\left({r}_{1}\right))$$

With this new set of criteria, the process was restarted. As a threshold for consensus, it was checked weather the number of items which fulfilled $\:Dt{M}_{{i}_{Ec}\left({r}_{n}\right)}\le\:0.1\stackrel{-}{6}$ were more than 75%. This threshold of 75% has been derived from existing literature and also used, for instance, by Ahmad et al.^102^, Alqahtani and Noman^103^, Chang and Hsu^59,60^, Chu and Hwang^113^, Hasim et al.^104^, Hsu and Chang^66^, Pua et al.^107^, Rahayu and Wulandari^108^, Rejab et al.^109^, Yusoff et al.^110,111^. Many of the aforementioned publications go back to the definition of this value by Murry and Hammons^91^. In addition to the numerous publications that employ this value, Diamond et al.^114^ investigated the median value of 75% in their widely cited delphi review study. This finding further substantiates the application of this value, as it is derived from a substantial body of research. If this threshold was not reached, a further round of reevaluation within the delphi panel was conducted.

Furthermore, the mean and median of the standard deviations and the interquartile ranges were considered^115,116^. They had the same threshold ($\:0.1\stackrel{-}{6}$) as previously defined. Employing these additional metrics in addition to the distance to mean enhances the rigor of the methodological approach by ensuring that consensus is generated not only through reliance on a single statistical metric, but through the integration of three distinct metrics^116^. The following literature references, identified by Gracht^116^, demonstrate the common application of the interquartile range^117–125^ and the standard deviation^126–128^ in delphi studies. In essence, the implementation of these two additional metrics was intended to provide supplementary support to the distance to mean.

### Fuzzy delphi criteria-based technology evaluation

To achieve a criteria-based evaluation of the different technologies ($\:t\in\:\left[1;8\right]$ as documented in Table A.1), another delphi panel was initiated. Here, the experts rated within a matrix of criteria and technologies each field with the evaluation scale shown in Table 5. We have applied a seven-point evaluation scale, as already deducted in the “Fuzzy delphi criteria weighting” section. The linguistic variables were adjusted, as shown in Table 5 since the performance of technologies for different criteria is the subject under consideration in this section.

**Table 5:** Evaluation scale for criteria-based technology evaluation.

Evaluation scale (linguistic variables)	Fuzzy numbers			Center of gravity
	$\:{f}_{Ect1}$	$\:{f}_{Ect2}$	$\:{f}_{Ect3}$	
Very poor performance	0.0	0.0	0.1	0.0333
Mostly poor performance	0.0	0.1	0.3	0.1333
Somewhat poor performance	0.1	0.3	0.5	0.3000
Neither good nor poor performance	0.3	0.5	0.7	0.5000
Somewhat good performance	0.5	0.7	0.9	0.7000
Mostly good performance	0.7	0.9	1.0	0.8667
Excellent performance	0.9	1.0	1.0	0.9667

From this point we applied the same procedure as already explained in the “Fuzzy delphi criteria weighting” section, which is why the following explanation is kept brief.

With 13 experts (see Table 3), 16 criteria (see Table D.1) and 8 technologies (see Table A.1), there are $\:{i}_{Ect}\left({r}_{1}\right)=1664$ items.

The $\:DtM$ values were calculated as outlined in Eq. (4) and filtered according to the same threshold as described in the “Fuzzy delphi criteria weighting” section ($\:Dt{M}_{{i}_{Ect}\left({r}_{n}\right)}\le\:0.1\stackrel{-}{6}$).

4 $$\:Dt{M}_{{i}_{Ect}\left({r}_{1}\right)}=\sqrt{\frac{1}{3}\left({\left({f}_{Ect{1}_{{r}_{1}}}-\frac{\sum\nolimits_{c=1}^{16}{f}_{Ect{1}_{{r}_{1}}}}{16}\right)}^{2}+{\left({f}_{Ect{2}_{{r}_{1}}}-\frac{\sum\nolimits_{c=1}^{16}{f}_{Ect{2}_{{r}_{1}}}}{16}\right)}^{2}+{\left({f}_{Ect{3}_{{r}_{1}}}-\frac{\sum\nolimits_{c=1}^{16}{f}_{Ect{3}_{{r}_{1}}}}{16}\right)}^{2}\right)}$$

After this, a reevaluation process was also initiated, resulting in a set of criteria for $\:{r}_{2}$ (see Eq. 5).

5 $$\:{i}_{Ect}\left({r}_{2}\right)=({i}_{Ect\_ev}\left({r}_{1}\right)+({i}_{Ect}\left({r}_{1}\right)-{i}_{Ect\_re}\left({r}_{1}\right))$$

The threshold for consensus and, subsequently, the initiation of further rounds follows the same procedure described in the “Fuzzy delphi criteria weighting” section.

### Final result generation

After the criteria were weighted (see “Fuzzy delphi criteria weighting” section) and the technologies underwent a criteria-based evaluation (see “Fuzzy delphi criteria-based technology evaluation” section), the final score for each technology $\:({S}_{t}$) was generated. For this, the according item set of the round which achieves consensus regarding the criteria importance and the criteria-based technology evaluation was taken. Since those two delphi panels run independently, the required number of rounds may vary, which is why in Eqs. (6), (7) and (8), we generally refer to the item set from the final round $\:{r}_{f}$. As a pre-step the $\:CoG$ vales for the criteria ($\:Co{G}_{{i}_{Ec}\left({r}_{f}\right)}$) and the technology ($\:Co{G}_{{i}_{Ect}\left({r}_{f}\right)}$) evaluation were multiplied for each criterion (see Eqs. 6 and 7). The center of gravity is a method that is employed for the purpose of defuzzification. We elected to implement the simple center of gravity method in this instance, a technique that has also been utilized by Hsu et al.^65^ and Wang et al.^98^.

In addition to the simple center of gravity approach, it is important to note that alternative defuzzification methods may lead to variations and thus potentially influence the final ranking outcomes. Methods such as the mean of maxima focus on specific characteristics of the membership function and may yield results that differ from those obtained through the center of gravity, which considers the entire distribution of membership degrees. In a similar manner, weighted average approaches assign varying degrees of importance to selected points. Consequently, these approaches can emphasize particular regions of the fuzzy set. Within the broader framework of center of gravity methods, a range of formulations exists. For instance, certain variants assign greater weight to the central value of a triangular fuzzy number by counting it twice, whereas the simple center of gravity method applied in this study treats all defining points equally, corresponding to an equal weighting scheme. To acknowledge the variety of defuzzification approaches, a sensitivity analysis was conducted in the “Sensitivity analysis” section.

6 $$\:Co{G}_{{i}_{Ec}\left({r}_{f}\right)}=\frac{1}{3}\left({f}_{Ec{1}_{{r}_{f}}}+{f}_{Ec{2}_{{r}_{f}}}+{f}_{Ec{3}_{{r}_{f}}}\right)$$

7 $$\:Co{G}_{{i}_{Ect}\left({r}_{f}\right)}=\frac{1}{3}\left({f}_{Ect{1}_{{r}_{f}}}+{f}_{Ect{2}_{{r}_{f}}}+{f}_{Ect{3}_{{r}_{f}}}\right)$$

For example, the CoG for “Adaptability” was multiplied with the CoG for “Adaptability of Laser”. To obtain final numerical values ranging from 0 to 1, it is necessary to multiply the $\:Co{G}_{{i}_{Ect}\left({r}_{f}\right)}$ by the relative value of the weighting $\:\left(\frac{Co{G}_{{i}_{Ec}\left({r}_{f}\right)}}{\sum\nolimits_{c=1}^{16}Co{G}_{{i}_{Ec}\left({r}_{f}\right)}}\right)$. The combination between criteria and technology results in 128 distinct mean values, in Eq. (8) referred to as $\:ct\in\:\left[1;128\right]$. Since each rating has a different distance to mean, the items which are presented for reevaluation differ for each expert. Then, all scores are summed up for each individual technology, creating eight distinct final scores ($\:{S}_{t}$) for the eight technologies under evaluation (see Eq. 8). Therefore, the final ranking is derived using a linear additive weighted model, which assumes full compensability among the selected criteria. This assumption is further discussed in the “Limitations” section. 

8 $$\:{S}_{t}=\sum\limits_{ct=1}^{128}\left(Co{G}_{{i}_{Ect}\left({r}_{f}\right)}\cdot \frac{Co{G}_{{i}_{Ec}\left({r}_{f}\right)}}{\sum\nolimits_{c=1}^{16}Co{G}_{{i}_{Ec}\left({r}_{f}\right)}}\right)$$

## Results and discussion

Since this article is focused on evaluating disassembly technologies for EOL EVBs, we have already shown the outcome of our stakeholder selection within the methods section (“Stakeholder selection”), which provided the basis for our further methodological steps. Therefore, in the following subsections, we concentrate on the results from the criteria identification, criteria weighting, and technology evaluation process.

### Evaluation criteria

A corpus of 16 EOL EVB disassembly technology evaluation criteria was identified through 65 expert interviews with relevant stakeholders (see “Criteria identification” section):

- Adaptability.
- Flexibility.
- Automatability.
- Capex.

(Capital expenditure)

- Opex.

(Operational expenditure)

- Digital compatibility.
- Environmental impact.
- Integratability.
- Process robustness and reliability.
- Productivity.
- Purity of outfeed.
- Qualification and availability of personnel.
- Repurposability.
- Safety.
- Scalability.
- Technology readiness.

The identified criteria, along with the experts attesting to their presence, are presented in Table D.1. It can be seen from Table D.1 that we consolidated the outcome of the expert interviews into the overarching 16 criteria. This consolidation step was conducted since we performed a delphi panel with a benchmark of disassembly technologies among those criteria later in this study. The higher the number of criteria, the more complex the delphi panel gets, so we wanted to reduce and consolidate the criteria based on the expert interviews. The consolidation process was conducted and iteratively discussed by the authors, who categorically grouped similar criteria. Table D.1 explicitly delineates the criteria mentioned by the respective expert. Furthermore, Table D.1 details the elements from the interviews that were ultimately consolidated into the 16 categories of criteria, thereby ensuring a transparent process from the initial interview contents to the final criteria employed in this study.

With the objective of enhancing methodological rigor, the consolidation process incorporated investigator triangulation. The review and interpretation of the interview material was carried out by multiple authors or instructed researchers, each working independently. This approach enabled the consideration of a variety of perspectives while reducing the potential for individual bias. The interpretations, which had been derived independently, were subsequently compared and aligned through discussion. Furthermore, a consensus-based validation approach was employed. Discrepancies in the grouping and interpretation of criteria were explicitly discussed among the authors until agreement was reached, ensuring intersubjective consistency without relying on formal coding procedures.

Moreover, the study ensures transparency through a clear audit trail and traceability of the consolidation process. Table D.1 provides a detailed mapping between the original interview statements and the final set of criteria, allowing readers to trace how individual inputs were abstracted and aggregated. This contributes to the overall reproducibility and credibility of the results. The robustness of the identified criteria is further substantiated by the considerable sample size and the principle of data saturation. The incorporation of 65 expert interviews serves to reduce the probability of omitting relevant criteria, while the increasing recurrence of themes across interviews indicates a comprehensive coverage of the domain. Furthermore, a limited number of criteria are necessary to ensure feasibility, cognitive manageability, and response quality within expert panels.

The consolidation thus reflects a deliberate balance between completeness and practical applicability. In addition, the iterative engagement with domain experts across both stages of the study reinforces the relevance and appropriateness of the selected criteria. This dual anchoring further reinforces the validity of the overall evaluation framework.

Along with the expert-based identification, we further enriched our criteria identification with references from the literature, as described in the “Technology selection” section and shown in Table D.1.

### Criteria weighting

The criteria identified in the “Criteria identification” section, particularly those outlined in Table D.1, were subjected to a weighting process conducted by experts in accordance with the methodology delineated in the “Fuzzy delphi criteria weighting” section. This weighting process was conducted with the delphi panel of 13 experts, as characterized in the “Fuzzy delphi criteria weighting” section. The final results of the weighting process are reported in Table F.1.

The results depicted in Table 6 reflect the outcome of round 2, as the expert panel has reached a consensus after two iteration rounds. Table 6 illustrates the statistical characteristics that indicate consensus. All relevant expert ratings are attached to this article in Appendix E.

**Table 6:** Statistical indicators for criteria weighting results.

Round	Percentage of items reaching the threshold for distance to mean	Mean (median) value of distance to means	Mean (median) value of the standard deviation of all criteria	Mean (median) value of the interquartile range of all criteria
Round 1	73.08%	0.1419 (0.1117)	0.1681 (0.1764)	0.2000 (0.1667)
Round 2	88.94%	0.0954 (0.0717)	0.1115 (0.1073)	0.0895 (0.1127)

As delineated in the methods section, the methodology incorporates established thresholds for attaining consensus among the expert panel. However, it is imperative to emphasize that attaining robust results among the various experts and across the multiple iteration rounds within the panel is equally crucial. Consequently, an analysis of the change in opinion between rounds 1 and 2 was conducted (see Table F.3). In Table F.3, the term “stage” is employed to denote the extent to which an expert’s opinion has shifted during the reevaluation process, with one stage representing a one-point change on the evaluation scale. Further analysis entails examining change in tendency, encompassing both scenarios where a shift towards or away from neutral is observed. In the first scenario, such a shift is interpreted as a change in tendency, while in the second scenario, it is not.

The subsequent discussion and interpretation of the results are conducted in the “Sensitivity analysis” section.

### Criteria-based technology evaluation

The final technology evaluation, executed in accordance with the methodological approach delineated in the “Fuzzy delphi criteria-based technology evaluation” section, yielded the results presented in Table F.2. These results reflect the outcome of the second round of the delphi panel, as this was the point at which consensus was reached. The exact ratings from all experts from both rounds are precisely documented in Appendix E.

The statistical parameters that indicate consensus after the second round are reported in Table 7.

**Table 7:** Statistical indicators for criteria-based technology evaluation results.

Round	Percentage of items reaching the threshold for distance to mean	Mean (median) value of distance to means	Mean (median) value of the standard deviation of all criteria	Mean (median) value of the interquartile range of all criteria
Round 1	59.98%	0.1680 (0.1314)	0.2030 (0.2120)	0.2474 (0.2000)
Round 2	79.33%	0.1174 (0.0853)	0.1456 (0.1418)	0.1557 (0.1667)

As performed for the criteria weighting in the “Criteria weighting” section, we also compared the results from round 1 and round 2, as shown in Table F.4.

A further discussion and interpretation of the results is performed in the discussion and the “Sensitivity analysis” section.

### Final weighted disassembly technology evaluation

In the final step, the defuzzied criteria-based disassembly technology evaluation was performed with the criteria weightings outlined in the “Final result generation” section. This resulted in the final ranking of EOL EVB disassembly technologies, which is presented in Table 8.

**Table 8:** Final technology ranking.

Technology	Final rank	Final score $\:{S}_{t}$
Laser	1	0.7577
Shredding	2	0.7012
Shear / Knife	3	0.6795
Milling	4	0.6590
Rotary cutting tool	5	0.6468
Robotic unscrewing	6	0.6010
Toolless manual removal	7	0.5678
Water jet	8	0.5560

### Sensitivity analysis

A comprehensive sensitivity analysis has been conducted to assess the robustness and stability of the results obtained from the fuzzy delphi-based technology evaluation. Given the inherent subjectivity in expert-based assessments, the objective of this analysis is to evaluate how variations in key inputs and assumptions influence the final outcomes. Specifically, the following four dimensions were examined: (1) variations in criterion-level expert judgments, (2) the influence of individual experts, (3) the choice of defuzzification method, and (4) the impact of DtM threshold and aggregation parameters used in the consensus measurement. The following discourse addresses the aforementioned four dimensions. The supplementary materials for the sensitivity analysis are reported in Appendix G.

In order to evaluate the sensitivity of the final technology rankings to changes in criteria weights, a systematic perturbation analysis was performed at the criterion level. For each of the 16 evaluation criteria, the aggregated expert assessment from the second (final) round was individually varied. Specifically, the expert ratings were adjusted by ± 1 and ± 2 for one single criterion on the underlying linguistic scale across all experts while holding all other criteria constant. The variation of ± 1 and ± 2 levels on the scale indicated in Table 4 is meant by this. This approach enables the isolation of each criterion’s influence on the overall results, facilitating the identification of criteria with a significant impact on the final ranking. By observing changes in technology scores and rankings under these controlled variations, the robustness of the evaluation against shifts in expert opinion can be assessed.

The results of this analysis are reported in Tables G.1–G.4. As demonstrated in Table G.2 and G.3, the findings reveal that there has been no change in the ranking for the rating variations of ± 1. This outcome is indicative of the negligible sensitivity exhibited by the present setup with respect to minor alterations in criteria weights. A subsequent analysis of variations in Table G.1 and G.4, with even deviations of ± 2, reveals only slight alterations in the ranking. The importance variation of + 2 for digital compatibility demonstrates a change in rank 7 and 8 (see Table G.1), indicating a certain degree of sensitivity for this criterion. The aforementioned observations also apply to the variation in the importance of the criteria, with a -2 adjustment given to Capex, Environmental impact, Purity of outfeed, and Repurposability. However, a variation of ± 2 on the scale indicated in Table 4 is also a substantial variation, leading to a minor change in the last two ranks of the ranking. This sensitivity is deemed acceptable. Furthermore, the variation of ± 1 did not result in any alteration in the ranking, thereby providing additional evidence that validates the robustness of the findings.

A leave-one-out analysis was conducted to examine the dependence of the results on individual expert opinions. In this procedure, each of the 13 experts was sequentially excluded from the dataset. The final results were then generated once more, this time with the exclusion of the aforementioned expert. This analysis offers insight into whether specific experts exert a dominant influence on the results. The results of this analysis are reported in Table G.5, indicating a change in the ranking for the exclusion of Expert 3. However, as previously observed in the variation of the criterion importance, only the final two ranks demonstrated change through this process, indicating a negligible impact, and thus, an acceptable level of sensitivity. This further substantiates the robustness of our study in relation to the evaluation of individual experts.

As defuzzification constitutes a pivotal phase in the fuzzy delphi method, an investigation was conducted into the sensitivity of results to the selected defuzzification method. The initial analytical framework employed the CoG method, as delineated in the “Final result generation” section. In order to assess the robustness of this selection, three common alternative defuzzification techniques were implemented. The mean of maximum (MoM) (see Eqs. 9 and 10), the signed distance method (SDM) (see Eqs. 11 and 12), and the graded mean integration representation (GMIR) (see Eqs. 13 and 14) were utilized. The following equations represent the three methods under consideration. It is imperative to note that one equation is employed for the defuzzification of the evaluation of the criteria importance, and another equation is utilized for the evaluation of the performance of each technology in relation with each criterion. This approach is analogous to Eqs. (6) and (7).

9 $$\:{MoM}_{{i}_{Ec}\left({r}_{f}\right)}={f}_{Ec{2}_{{r}_{f}}}$$

10 $$\:{MoM}_{{i}_{Ect}\left({r}_{f}\right)}={f}_{Ect{2}_{{r}_{f}}}\:$$

11 $$\:{SDM}_{{i}_{Ec}\left({r}_{f}\right)}=\frac{1}{4}\left({f}_{Ec{1}_{{r}_{f}}}+2\cdot{f}_{Ec{2}_{{r}_{f}}}+{f}_{Ec{3}_{{r}_{f}}}\right)$$

12 $$\:{SDM}_{{i}_{Ect}\left({r}_{f}\right)}=\frac{1}{4}({f}_{Ect{1}_{{r}_{f}}}+2\cdot{f}_{Ect{2}_{{r}_{f}}}+{f}_{Ect{3}_{{r}_{f}}})$$

13 $$\:{GMIR}_{{i}_{Ec}\left({r}_{f}\right)}=\frac{1}{6}\left({f}_{Ec{1}_{{r}_{f}}}+4\cdot{f}_{Ec{2}_{{r}_{f}}}+{f}_{Ec{3}_{{r}_{f}}}\right)$$

14 $$\:{GMIR}_{{i}_{Ect}\left({r}_{f}\right)}=\frac{1}{6}({f}_{Ect{1}_{{r}_{f}}}+4\cdot {f}_{Ect{2}_{{r}_{f}}}+{f}_{Ect{3}_{{r}_{f}}})$$

By comparing the resulting technology scores and rankings across these methods, the analysis evaluates whether the study’s conclusions are dependent on the specific defuzzification approach or remain. The outcomes for this are reported in Table G.6 and show no variation in the final ranking, indicating no significant sensitivity regarding the defuzzification approach, further substantiating the robustness of our approach.

The final stage of the sensitivity analysis entailed the examination of the sensitivity of the consensus measurement. This was achieved by varying key parameters that were used in the fuzzy delphi process. The initial analysis implemented a threshold value of $\:\le\:0.1\stackrel{-}{6}$ for the DtM and other statistical parameters, which serves to determine whether consensus is achieved (see “Fuzzy delphi criteria weighting” and “Fuzzy delphi criteria-based technology evaluation” sections). In order to conduct a sensitivity analysis, the threshold was adjusted to 0.20, a value that has been frequently cited in the extant literature (see “Fuzzy delphi criteria weighting” section). As demonstrated in Tables 6 and 7, all statistical parameters, including the DtM, were found to be $\:\le\:0.1\stackrel{-}{6}$. This indicates that they were also $\:\le\:0.2$ for round 2, thereby demonstrating that there was no difference in the process, irrespective of the threshold employed. Furthermore, in the initial round, values greater than 0.2 were observed in Table 7. This necessitated the implementation of a second round for both DtM values. Consequently, the alteration of the DtM value would have exhibited no impact on the procedure, thereby demonstrating no sensitivity to the results of this study regarding this threshold value.

Moreover, the calculation of the DtM and other statistical measures was diversified by using the mean and the median (see Tables 6 and 7). In this study, both the mean and median were incorporated as necessary requirements, thereby enhancing the robustness of the model.

Overall, the sensitivity analysis demonstrates the model’s robustness across various dimensions. The observed changes were of a minor nature, affecting only the final two ranks of the original ranking, which were in close proximity. However, observed sensitivities are indicated in this section, which should be considered in further investigations.

### Discussion

The methodological approach proposed in this study can serve as a blueprint for other studies, particularly in the context of technology evaluation, where the initial stages of criteria identification and selection usually tend to be less systematic. Within the scope of our study, a systematic approach was adopted for the selection of stakeholders, serving as a preliminary stage for the subsequent expert selection and criteria identification and evaluation process. In our case, all relevant stakeholders contributed to the criteria base, offering their unique perspectives. However, we restricted the technology evaluation and criteria weighting to experts with extensive knowledge in the field, ensuring valid ratings. This approach is further validated by the delphi panel’s consensus after only two rounds despite the use of comparatively stringent thresholds. As illustrated in Tables 6 and 7, the statistical metrics employed in this study were more extensive in scope compared to those of other studies. This is due to our consideration of additional variables, including the distance to the mean, the standard deviation, and the interquartile range. The necessity to reach our predetermined threshold for all these metrics serves to reduce the random dependency on a single measure. This approach can serve as a blueprint for future studies.

A notable distinction emerged among experts during the reevaluation process in round 2 (see Tables F.3 and F.4). The degree to which experts modify their initial opinions indicates the extent to which they are open to alternative perspectives. This phenomenon is influenced by their personal attitudes and openness to new arguments, as well as potential information asymmetries. Information asymmetries can emerge even among delphi panel experts engaged in EOL EVB disassembly, given their diverse professional backgrounds and technological experiences. The study results indicate that the percentage of items requiring reevaluation in the second round is significantly higher for the technology evaluation than for the criteria weighting. This observation is consistent with the results for the criteria weighting, as almost all items were rated as high importance. This suggests that the initial criteria identification process developed was effective, as the experts generally rated the criteria as important. Safety and process robustness and reliability have been identified as particularly important topics, with a CoG > 0.9. This underscores the high risk associated with handling EOL EVBs, particularly in the context of dismantling, which necessitates consideration in future technological developments, particularly at the machine and system level. The high ratings assigned to process robustness and reliability indicate that experts are already contemplating the industrialization of this process, where such topics are of paramount importance. This is followed by productivity and costs (Capex/Opex), flexibility and adaptability, scalability and technology readiness, and purity of outfeed with a CoG of > 0.8. These metrics also indicate that the experts already consider the practical challenges of implementing mass-capable systems, suggesting high development and deployment pressure. Repurposability was regarded as comparably unimportant by a significant margin. The prevailing viewpoint among experts in the field is that this subject is predominantly academic in nature and is not yet economically viable. Additionally, significant unresolved issues, such as liability and insurance, can be highlighted as key challenges.

The comparatively elevated proportion of items necessitating reevaluation in the technology evaluation can be attributed to the varying degrees of expertise and potential biases experts hold concerning the technologies in question, resulting in a spectrum of perspectives. The significant number of items requiring reevaluation in the technology evaluation (averaging 40%) further substantiates implementing the iterative fuzzy delphi method with a consensus objective instead of conventional expert ratings. Additionally, the experts exhibited a notable degree of openness to modifying their perspectives in the second round (averaging up to 70%). This observation underscores the notion that even experts show uncertainty in specific domains and can adapt their perspectives. This further substantiates the efficacy of our fuzzy delphi approach, wherein fuzzy logic is employed as a metric to quantify uncertainty. Furthermore, as determined by statistical investigations, the consensus was consistently higher for the criteria weighting compared to the technology evaluation. This discrepancy can potentially also be attributed to the influence of expert background and bias. Additionally, the extensive pre-process for criteria identification may also have contributed to the robustness of the criteria weighting. Furthermore, the statistical investigation observed a notable discrepancy between the mean and median values. Therefore, it is recommended that future studies consider both values in their analyses.

The findings of our evaluation of disassembly technologies for EOL EVBs indicate that laser technology emerges as a promising approach, not only in comparison to other semi-destructive technologies such as milling or shear/knife cutting but also in relation to destructive technologies such as shredding. This outcome underscores the necessity for further research in laser-based disassembly approaches.

The results indicate a substantial gap between laser and shredding technology. Key factors contributing to the superior performance of laser technology include the low purity of shredded materials, the inability to reuse components, the significant environmental impact, and the high operational expenses associated with shredding. This suggests that the shredding of entire battery packs may not be the optimal approach, as it has been identified as a contributing factor to the impurity levels associated with the shredding operations. Consequently, the disassembly to module or even cell level could potentially enhance the rating of the shredding technology. This process has also the potential to expand the scope of reuse for components other than battery modules or cells, thereby addressing the current low rating concerning the reuse criterion for shredding. In this regard, a coexistence between laser-based disassembly and shredding could be a beneficial approach for integrating both technologies. This approach would involve focusing on the disassembly of battery packs at the pack or module level rather than shredding the battery pack level.

However, the implementation of laser technology in the disassembly of EOL EVBs is also confronted by substantial barriers. For instance, the issue of safety is of particular pertinence in the context of laser cutting, a process that is inherently associated with thermal separation mechanisms^129^. The thermal influence of the laser cutting process has the potential to damage the battery cells and restrict their reusability. Furthermore, safety hazards may emerge during the disassembly process, particularly when executing laser cuts. For instance, the presence of electrolyte contamination within the battery pack can result in the occurrence of a flammable area. Moreover, the considerable Capex required for laser technology could hinder its application in the price-sensitive domain of recycling. In addition to the considerable initial investment, companies must also ensure the training of workers in the operation of laser equipment, which adds to the initial expenses associated with implementing this technology. Nevertheless, there has been a marked decline in global laser prices towards a commoditization, a development that has the potential to render the technology more affordable in the future^130^. Furthermore, advancements are being made in the realm of training opportunities. These developments encompass not only the field of laser technology but also the broader integration of asynchronous learning methods, such as online learning. In the subsequent ranking, the other semi-destructive technologies are closely grouped, except water-jet cutting. Non-destructive disassembly technologies were also found to be closely ranked. Water-jet cutting received the lowest score due to its high wastewater generation, potentially hazardous materials involvement, component wetness, subsequent reuse, and further processing limitations.

### Implications

This section synthesizes the theoretical, practical, and managerial implications derived from the study and its methodological framework.

From a theoretical perspective, this research establishes a robust foundation for evaluating early-stage technologies through a structured and stakeholder-oriented criteria development process. The theoretical framework presented in this study represents a foundational contribution, as it introduces a systematic methodology for generating evaluation criteria grounded in stakeholder input. A notable advancement lies in the explicit and transparent approach to stakeholder identification and selection, which enhances the validity and relevance of the resulting criteria set and can be considered a distinct theoretical contribution to technology management research.

Furthermore, the study advances the fuzzy delphi methodology by introducing a novel and logically derived approach to the calculation of the degree of consensus. Rather than relying on established threshold values from prior literature, this research develops an internally consistent derivation and validates it within a case study context. Furthermore, methodological rigor is enhanced through the combined use of multiple statistical indicators to determine the necessity of additional delphi rounds. This multi-metric approach enhances the robustness of the decision rules when compared to conventional single-metric approaches. Consequently, it contributes to the extant body of research on decision-making in technology management, particularly in contexts characterized by high uncertainty and limited experimental data.

From a practical standpoint, this study provides the first comprehensive and systematic benchmarking of EOL EVB disassembly technologies using a structured multi-criteria methodology. Beyond serving as an application case, several elements of the study provide direct value for practitioners. The developed criteria set offers a ready-to-use evaluation framework that can be adapted to benchmark additional disassembly technologies or to reassess given technologies as they evolve over time. This enables practitioners to update decision bases in accordance with technological progress and evolving market conditions.

Furthermore, the stakeholder analysis provides actionable insights by identifying relevant actors and perspectives that should be considered in technology evaluation and broader technology management processes. This initiative aims to assist organizations in the more effective structuring of their own stakeholder engagement. It is important to note that, while the methodology has been demonstrated in the context of disassembly technologies, the overall methodology constitutes a practical and validated approach that can be transferred to other early-stage technologies requiring structured evaluation. This framework is especially applicable to corporate technology and innovation management functions, as well as to units such as business development, where informed technological decision-making under uncertainty is paramount.

From a management perspective, the presented methodology addresses a fundamental challenge in early-stage technological decision-making. Organizations are obligated to make decisions that are both strategically significant and path-dependent at a point where reliable quantitative data, such as field or experimental data, are scarce or unavailable. Conversely, postponing such decisions can potentially lead to the loss of opportunities or a decline in competitiveness. This dynamic engenders a tension between uncertainty and the necessity for timely commitment.

The expert-based evaluation approach developed in this study provides a structured and transparent basis for decision-making under such conditions. The integration of expert knowledge enables management to derive well-founded, traceable, and communicable decisions despite limited data availability. This approach has been demonstrated to enhance not only the quality of decisions made, but also to promote organizational alignment, a phenomenon characterized by the explicit documentation of assumptions, evaluation logic and structured stakeholder involvement.

Furthermore, it facilitates the comparison of alternative technological pathways and helps prioritize investments in a resource-constrained environment. The structured evaluation process can also function as a medium for communication between technical and managerial stakeholders, thereby facilitating the alignment of engineering perspectives with strategic decision-making.

In conclusion, the approach is well suited to iterative application. This allows management to reassess decisions as new information becomes available or as technologies mature. This approach fosters adaptive decision-making and continuous strategy refinement, which are pivotal in dynamic technological environments. In essence, the methodology enhances an organization’s capacity to navigate early-stage technology landscapes with elevated levels of confidence, transparency, and strategic coherence.

The financial implications of this study primarily lie in its ability to support economically informed decision-making under conditions of high uncertainty, as previously outlined in the proposed methodology.

A central contribution of the present study is that the methodology translates expert-based assessments into economically relevant dimensions. This enables decision-makers to methodically integrate financial considerations even in the absence of reliable quantitative data, which is characteristic of early-stage technologies.

A significant implication of this approach pertains to the comparability of technological alternatives from a financial perspective. By consolidating multi-dimensional performance metrics into a unified evaluation framework, this approach facilitates relative positioning of technologies. This is particularly valuable in the nascent stages of a project, when traditional techno-economic analyses are often not feasible due to data limitations.

Moreover, the methodology enhances investment decision processes by improving traceability and justification. The structured evaluation results can be utilized by management to communicate and defend investment choices.

The framework is designed for iterative application, allowing assessments to be refined over time as more data becomes available. Initial qualitative evaluations can thus evolve into more detailed economic analyses, ensuring continuity in decision-making from early exploration to industrial implementation.

### Deployment recommendations

This section provides guidance for deploying the proposed framework in industrial contexts, clarifies boundary conditions for its transferability, and explains its validation through a real-world case application.

The framework is particularly well-suited for early-stage technology evaluation, where quantitative data are limited but expert knowledge is available. The applicability of this model is contingent upon the consideration of several boundary conditions. Firstly, it is imperative to ensure access to a sufficiently diverse and independent expert panel, ideally covering multiple perspectives along the value chain. Secondly, the candidate technologies must be comparable within a shared system boundary and evaluable through a consistent set of criteria. Finally, transparency and process governance, such as traceability of criteria development and anonymity in expert consultation, are necessary to ensure validity and reduce bias.

Within these boundaries, deployment must follow a structured and replicable process aligned with corporate technology management practices. The initial phase encompasses scoping and governance definition, incorporating the clarification of decision objectives, system boundaries, and responsibilities. Notably, the framework necessitates predefined decisions concerning linguistic scales, consensus thresholds, and aggregation methods, which can be calibrated to the particular requirements of the specific application context. Sensitivity analysis should be an integral part of deployment, allowing decision-makers to test the robustness of results against variations in key assumptions, expert composition, and methodological parameters.

The present study itself serves as a real-world case application of the framework in the context of EOL EVB disassembly. The methodology was implemented in its entirety, encompassing stakeholder-driven criteria development, fuzzy delphi-based weighting, systematic technology benchmarking, and extensive sensitivity analysis. The results, which are documented in the “Results and discussion” section and the supplementary materials, demonstrate the practical feasibility and internal consistency of the approach.

In summary, while the proposed methodology is not universally applicable, it is broadly deployable under defined conditions and with moderate adaptation. It provides a structured pathway for translating expert knowledge into transparent and robust technology evaluations and has been validated through a comprehensive real-world application in the field of EOL EVB disassembly.

### Limitations

This study is subject to several limitations, the details of which are elaborated in the following. The technologies selected for the benchmark were based on a previous study grounded on a literature analysis. It is, therefore, possible that companies are developing disassembly technologies that are not publicly documented. Consequently, there is a possibility that we have omitted certain technologies. Furthermore, disassembly technologies were evaluated in a rather general manner, encompassing separation operations during disassembly. This is a focused field of research. However, future studies could apply the proposed methodology to even a narrower field of application, such as evaluating disassembly technologies on a specific sub-step of the EOL EVB disassembly. For this, they can apply the given methodology.

Despite the measures taken to ensure a diverse and balanced expert panel, the possibility of technological bias cannot be entirely excluded. Experts’ judgments may inherently be influenced by their individual professional backgrounds, prior experiences, and familiarity with specific technologies. Such biases are a known limitation in delphi-based studies, particularly when evaluating emerging or specialized technological fields. In the context of this study, particular attention must be given to a potential bias toward laser-based disassembly technologies, which achieved the highest ranking in the evaluation. While this outcome is supported by the aggregated expert assessments, it may be partially influenced by individual expert perspectives. Notably, only Expert 7 out of the 13 experts is directly affiliated with the laser technology sector. This suggests that while a bias may exist at the individual level, its overall influence on the aggregated results is likely limited due to the small proportion within the panel. Nevertheless, the possibility that this affiliation may have contributed to a more favorable assessment of laser technologies cannot be fully ruled out. However, the sensitivity analysis in the “Sensitivity analysis” section does not show any changes in the ranking for the leave-one-out analysis of Expert 7.

A key limitation of the applied aggregation approach (see Eq. 8) lies in the use of a linear additive weighted model for deriving the final ranking, which inherently assumes full compensability among criteria. This implies that poor performance in one criterion can be completely counterbalanced by exceptional performance in another. While this assumption simplifies aggregation and enhances interpretability, it can be a limitation in safety-critical contexts, where certain criteria, such as safety and robustness, would be considered non-compensatory or only partially compensatory. In such cases, the allowance of trade-offs may result in rankings that underestimate critical shortcomings, potentially rendering unacceptable risks undetected. While the weighting scheme does in fact reflect the relative importance of the criteria to a certain extent, it does not eliminate the possibility of compensation effects. Consequently, the results of this study should be interpreted cautiously, particularly in contexts involving highly consequential safety-critical decisions. Subsequent studies may investigate alternative aggregation methods, including non-compensatory models (e.g., outranking approaches or threshold-based techniques). These methods could enhance the capture of decision logic in safety-critical environments and assess the robustness of ranking outcomes.

Additionally, the professional backgrounds of the experts may introduce a technological bias, which could influence their rating behavior. To ensure a balanced and comprehensive input, particular attention was directed towards ensuring a high degree of diversity among the selected experts. This encompassed not only the diversity of stakeholder groups but also the technological orientation. A potential bias towards specific disassembly technologies is primarily relevant for machine builders, as they tend to focus on particular technologies that align with their areas of expertise.

To address this, machine builders were selected from different disassembly technology domains to ensure that no single technological approach is overrepresented. This risk is less relevant in the other stakeholder groups. Recyclers, dismantlers, OEMs, and academic experts generally exhibit a significantly lower technology bias in this case, as their operational or scientific objectives require openness to the most promising and successful technologies. Consequently, the composition of the expert panel reflects a balanced distribution across industry and academia along the supply chain, in addition to an adequate representation of technological perspectives, thereby reducing the potential for biased outcomes. In addition, the personal attitudes and behaviors of the experts should be considered, as they have a significant impact on the results of the delphi study, given that this methodology is based on adjusting opinions.

### Future research and transfer to other domains

The developed methodology is especially beneficial in situations where the basis for informed decision-making is primarily expert opinion, which is a common occurrence in the initial stages of technology management. Proposed as a potential avenue for future research and a practical application, the methodology should be incorporated into a tool-based solution to facilitate its implementation in technology management decision-making processes. A pilot version of such a tool has already been developed and discussed within a company as a part of this study. In this context, the proposed application of the tool centers on its role as a pivotal instrument in the evaluation of emerging technologies in their early stages. Nonetheless, limitations of the tool’s applicability became apparent during its implementation. For instance, if concrete data regarding technologies are already established, particularly through preliminary trial studies. In this case, a purely expert-based approach is no longer sufficient, thereby limiting the applicability of the tool and the underlying framework. It is imperative that subsequent investigations examine the feasibility of integrating trial data and other forms of technology data into this expert-based approach. A hybrid methodology that encompasses these diverse data sources beyond the initial stages of technology development is a concrete pathway for future research.

From a technological perspective, future studies should prioritize advancing semi-destructive disassembly technologies, with a particular emphasis on laser-related approaches. A potential research direction for future studies is to examine the thermal impact of laser-based disassembly processes. Laser cutting is a thermal separation process that has the potential to cause damage to battery cells, which could result in fires. This would also address the safety evaluation of laser technology. Rettenmeier et al.^129^ initiated the first investigations in this direction, however, the findings must undergo validation by experimental studies.

From an analytical perspective, future research can build upon the expert evaluation data presented in this study to conduct further analyses. For example, a correlation analysis could identify potential redundancies, which may be eliminated to reduce the effort required for subsequent investigations in this domain. In addition to examining correlations within expert ratings, analyses could also explore potential interdependencies between technologies and criteria. Furthermore, stakeholder groups could be analyzed for correlation patterns, potentially revealing information asymmetries or highlighting areas requiring further discussion.

Finally, the proposed methodology should be applied to additional technological domains for the purpose of evaluating emerging technologies in the early stages.

## Conclusions

This study has introduced a robust fuzzy delphi-based methodology for evaluating emerging technologies in their early stages. By integrating fuzzy logic with expert consensus, our approach bridges the gap between the inherent uncertainty of early-stage innovation and the need for systematic decision-making. In addressing the guiding research question, the study demonstrates the feasibility of systematically assessing early-stage emerging technologies using techno-economic criteria. The findings indicate that a methodical integration of stakeholder-driven criteria development, fuzzy delphi-based weighting, and comparative technology benchmarking facilitates a transparent, reproducible, and decision-relevant evaluation, even in contexts characterized by limited quantitative data and high uncertainty. This methodological framework significantly expands the current state of research by offering a granular process that can be adapted to various technological domains under certain boundary conditions. A significant contribution of this work is developing a novel method for criteria identification, which establishes a solid foundation for constructing a technology benchmark. The practical application of our methodology was demonstrated through a detailed case study on the disassembly of EOL EVBs. In this instance, the systematic evaluation process successfully identified laser cutting and shredding as the most promising technologies. This outcome underscores the potential of the proposed approach to provide clear, actionable insights, thereby enhancing the strategic prioritization of technological development efforts. The case study validates the methodology and illustrates its capacity to generate widely accepted benchmarks that facilitate informed decision-making in technology management. In essence, our contributions lie in furnishing the battery industry with a comprehensive decision-making framework, thereby facilitating the advancement of disassembly technologies.

## Electronic Supplementary Material

Below is the link to the electronic supplementary material.

Supplementary Material 1 (Appendices A, B, C, D, F, G)

Supplementary Material 2 (Appendix E)

## Data Availability

All data supporting the findings of this study are available within the paper and its Supplementary Information / Appendices.
