# Supplementary material for: From blur to blueprint: a fuzzy delphi methodology for evaluating early-stage emerging technologies—the case of end-of-life automotive traction battery disassembly
Source: Sci Rep. 2026 May 19;16:15516. doi: 10.1038/s41598-026-50592-1 (PMC13187476; doi:10.1038/s41598-026-50592-1)
Supplement: Supplementary file 1 — Supplementary Material 1 (Appendices A, B, C, D, F, G) [file 41598_2026_50592_MOESM1_ESM.docx]

# Supplementary material

*From blur to blueprint: A fuzzy delphi methodology for evaluating early-stage emerging technologies – The case of end-of-life automotive traction battery disassembly*

Appendix A: Supplementary material for the technology identification 3

Table A.1: Disassembly technologies considered in this study 3

Appendix B: Supplementary material for the stakeholder identification 4

Table B.1: Search details in AI-tools (administration) 4

Table B.2: Search details in AI-tools (content) 4

Table B.3: Consolidated comparison of AI-tools output 7

Table B.4: Results from stakeholder evaluation workshop according to the stakeholder salience model from Mitchell et al. [34] 8

Appendix C: Supplementary material for the criteria identification 10

Table C.1: Expert characterization for interviews to identify criteria 10

Appendix D: Supplementary material for the results 13

Table D.1: Evaluation criteria from the expert interviews 13

Appendix E: Expert ratings from the delphi panel 16

Appendix F: Aggregated results from the delphi panel 17

Table F.1: Results of criteria weighting process 17

Table F.2: Results of criteria-based technology evaluation process 17

Table F.3: Change metrics between round 1 and 2 for criteria weighting 22

Table F.4: Change metrics between round 1 and 2 for criteria-based technology evaluation 24

Appendix G: Supplementary material for the sensitivity analysis 26

Table G.1: Sensitivity analysis for criterion importance variation (+2) 27

Table G.2: Sensitivity analysis for criterion importance variation (+1) 28

Table G.3: Sensitivity analysis for criterion importance variation (-1) 29

Table G.4: Sensitivity analysis for criterion importance variation (-2) 30

Table G.5: Sensitivity (leave-one-out) analysis for Experts 32

Table G.6: Sensitivity analysis for defuzzification method variation 33

References 34

## Supplementary material for the technology identification

### Disassembly technologies considered in this study

| Disassembly technology categorization [1] | Disassembly technologies [1] | Excerpt of references on selected disassembly technologies |
| --- | --- | --- |
| Destructive | Crushing (wet / dry) | [2]; [3]; [4] |
| Semi-destructive | Desoldering (excluded) | [5] |
| Semi-destructive | Drilling (excluded) | [6]; [7]; [8]; [9] |
| Semi-destructive | Knife / shear cutting | [2]; [6] |
| Semi-destructive | Laser cutting | [6]; [10]; [11]; [12]; [13]; [9]; [5]; |
| Semi-destructive | Milling | [6]; [7]; [14]; [15]; [16]; [9]; [17]; [18] |
| Semi-destructive | Oxy fuel cutting (excluded) | [6] |
| Semi-destructive | Plasma cutting (excluded) | [6] |
| Semi-destructive | Rotary cutting tools (incl. saw and angle grinder | [2]; [6]; [19]; [11]; [14]; [20]; [18] |
| Semi-destructive | Water-jet cutting | [6] |
| Non-destructive | Unscrewing | Screw detection: [21]; [22]; [23]; [12]; [17]; [18]  Process of unscrewing (including experimental setups): [24]; [25]; [26]; [27]; [28]; [19]; [29]; [30]; [13]; [31]; [32]; [18]; [33] |
| Non-destructive | Toolless manual removal or separation operations | [13]; [33] |

## Supplementary material for the stakeholder identification


### Search details in AI-tools (administration) (AI = Artificial Intelligence)

| AI-tool | Version | Prompt | Search time (date, time and time zone) |
| --- | --- | --- | --- |
| ChatGPT | GPT-4o | brainstorm a list of potential stakeholders for the area 'disassembly technologies of end-of-life automotive battery packs.' | 09/23/2024 at 10:43 (CEST) |
| Microsoft CoPilot | Free version (not specified) | brainstorm a list of potential stakeholders for the area 'disassembly technologies of end-of-life automotive battery packs.' | 09/23/2024 at 10:48 (CEST) |
| Perplexity AI | Free version (not specified) | brainstorm a list of potential stakeholders for the area 'disassembly technologies of end-of-life automotive battery packs.' | 09/23/2024 at 10:53 (CEST) |
| Google Gemini | Gemini 1.5 Flash | brainstorm a list of potential stakeholders for the area 'disassembly technologies of end-of-life automotive battery packs.' | 09/23/2024 at 13:19 (CEST) |

### Search details in AI-tools (content)

| AI-tool | Result (the response text of the tool without content was left out and formatting was standardized towards enhanced readability without changing the content of the responses) |
| --- | --- |
| ChatGPT | Automotive Industry   - Automobile Manufacturers (OEMs): Companies like Tesla, Toyota, Ford, GM, Volkswagen, etc., producing electric vehicles (EVs) with an interest in efficient battery disassembly for recycling or reuse. - Tier 1 and 2 Suppliers: Companies that supply parts and subsystems for EVs, including battery systems and components, may be directly involved in end-of-life disassembly technology. - EV Startups: Smaller, emerging companies working on electric vehicles may have specialized interests in managing battery lifecycle.   Battery Manufacturers   - Battery Cell Manufacturers: Companies like Panasonic, LG Chem, CATL, and others that manufacture lithium-ion cells used in EVs. They have a vested interest in the materials recovery process. - Battery Pack Assemblers: Entities that assemble battery cells into modules and packs may be involved in designing for disassembly.   Recycling & Reuse Industry   - Battery Recycling Companies: Firms like Li-Cycle, Redwood Materials, and Umicore that specialize in recovering valuable materials (e.g., lithium, cobalt, nickel) from used battery packs. - Material Recovery and Smelting Companies: Industries that extract critical materials from dismantled batteries for reuse in manufacturing. - Second-Life Battery Companies: Companies focused on repurposing used battery packs for stationary energy storage systems, requiring disassembly and reconfiguration.   Technology Providers   - Automation and Robotics Companies: Providers of robotic systems and automation technologies for the efficient, safe disassembly of battery packs. - Artificial Intelligence (AI) and Machine Vision Companies: Firms offering AI-driven solutions for identifying components, managing disassembly processes, and improving efficiency. - Disassembly Tool Manufacturers: Companies that develop specialized tools and technologies for manual or automated battery pack disassembly.   Environmental and Regulatory Bodies   - Government Agencies & Regulators: National and regional bodies responsible for setting and enforcing environmental regulations around battery disposal, recycling, and hazardous materials (e.g., Environmental Protection Agency (EPA), EU directives, etc.). - Standards Organizations: Groups like SAE International, ISO, or IEEE that establish standards and guidelines for battery disassembly and recycling processes. - Environmental NGOs and Advocacy Groups: Organizations focused on promoting sustainability, reducing waste, and minimizing the environmental impact of electric vehicle batteries.   Research Institutions and Universities   - Academic Research Groups: Researchers working on materials science, battery lifecycle management, and disassembly techniques. - Industry-Academic Partnerships: Collaborative research efforts between automotive companies, recyclers, and universities to develop new technologies or processes for battery disassembly.   Supply Chain Partners   - Raw Material Suppliers: Companies providing raw materials, such as lithium, cobalt, and nickel, have an interest in the recycling stream for securing material supply. - Logistics and Transportation Companies: Firms involved in transporting end-of-life battery packs to recycling or disassembly centers, ensuring safety and compliance with regulations.   End Users and Consumers   - EV Owners and Fleet Managers: Consumers and companies that need responsible disposal or recycling solutions for their vehicles' battery packs at end-of-life. - Public Utilities & Energy Storage Providers: These entities may use repurposed batteries for grid storage applications and thus may influence or participate in battery disassembly efforts.   Policy Makers and Legislators   - National/International Policymakers: Government officials responsible for drafting legislation around waste management, battery recycling mandates, and incentives for sustainable disassembly technologies. - Regional/Local Governments: Local authorities regulating disposal practices, land use, and environmental impact in their jurisdictions.   Insurance and Risk Management   - Insurance Companies: Firms that manage risks related to the transport, storage, and disassembly of hazardous materials like automotive batteries. - Risk Assessment and Compliance Auditors: Organizations that assess environmental, safety, and financial risks associated with battery disassembly processes.   Energy Industry   - Renewable Energy Companies: Companies looking to repurpose battery packs for renewable energy storage in solar or wind projects. - Power Grid Operators: Entities interested in second-life battery applications for balancing loads and enhancing grid storage.   Consumer Advocacy and Awareness Groups   - Consumer Watchdog Organizations: Groups that focus on product lifecycle transparency, ensuring companies adhere to sustainable practices in battery disassembly and recycling. - Sustainability and Circular Economy Advocates: Organizations and consumers pushing for environmentally friendly end-of-life solutions for EV batteries. |
| Microsoft CoPilot | - Automotive Manufacturers: Companies producing electric vehicles (EVs) and hybrid vehicles. - Battery Manufacturers: Firms that design and produce automotive batteries. - Recycling Companies: Businesses specializing in recycling and material recovery. - Government Agencies: Regulatory bodies overseeing environmental and safety standards. - Research Institutions: Universities and research centers focusing on sustainable technologies. - Environmental Organizations: NGOs and advocacy groups promoting eco-friendly practices. - Technology Providers: Companies developing disassembly and recycling technologies. - Logistics Providers: Firms handling the transportation of end-of-life batteries. - Energy Companies: Utilities and firms interested in second-life applications for batteries. - Investors and Venture Capitalists: Financial entities investing in green technologies. - Consumers: EV owners and the general public concerned with sustainability. - Industry Associations: Groups representing the interests of the automotive and battery industries. - Waste Management Companies: Firms managing the disposal and recycling of hazardous materials. - Policy Makers: Individuals and groups involved in creating regulations and policies. - Safety and Compliance Experts: Professionals ensuring adherence to safety standards during disassembly. |
| Perplexity AI | Manufacturers and Suppliers   - Automotive Manufacturers: Companies that produce electric vehicles (EVs) and their components, which need to manage battery end-of-life processes. - Battery Manufacturers: Producers of lithium-ion batteries and other types used in EVs, involved in designing for disassembly and recycling. - Component Suppliers: Suppliers of materials and components that make up battery packs, interested in recovery and reuse.   Research Institutions and Universities   - Academic Researchers: Institutions conducting research on disassembly technologies, recycling methods, and sustainability practices. - Technical Research Organizations: Entities like Fraunhofer IPA that develop innovative disassembly technologies and processes.   Recycling and Waste Management Companies   - Battery Recycling Firms: Companies specializing in the recycling of battery materials, focusing on recovering valuable resources. - Waste Management Services: Providers that handle the collection, transportation, and processing of end-of-life batteries.   Government and Regulatory Bodies   - Environmental Agencies: Government organizations that set regulations for battery disposal, recycling standards, and sustainability initiatives. - Policy Makers: Individuals or groups involved in creating policies supporting circular economy practices related to battery use.   Industry Associations   - Automotive Industry Associations: Groups that represent the interests of automotive manufacturers and advocate for sustainable practices. - Battery Industry Associations: Organizations focused on promoting advancements in battery technology and recycling initiatives.   Technology Developers   - Automation Technology Companies: Firms developing automated solutions for battery disassembly processes. - Software Developers: Companies creating software for managing disassembly operations, including tracking materials and optimizing processes.   Investors and Financial Institutions   - Venture Capitalists: Investors looking to fund innovative technologies in battery disassembly and recycling. - Banks and Financial Institutions: Entities providing financial support for projects aimed at improving battery lifecycle management.   Consumer Advocacy Groups   - Environmental NGOs: Organizations advocating for sustainable practices in battery production and disposal. - Consumer Rights Groups: Advocates focusing on informing consumers about the environmental impacts of battery waste. |
| Google Gemini | Industry Stakeholders   - Automotive Manufacturers: Tesla, Toyota, Volkswagen, General Motors, Ford, etc. - Battery Manufacturers: LG Chem, Panasonic, CATL, Samsung SDI, etc. - Recycling Companies: Retriev Technologies, Li-Cycle Corp, Redwood Materials, etc. - Raw Material Suppliers: Mining companies (e.g., lithium, cobalt, nickel), chemical manufacturers - Technology Providers: Companies specializing in disassembly equipment, robotics, AI, and material recovery - Government Agencies: Regulatory bodies, research institutes, and funding agencies   Academic and Research Institutions   - Universities: Departments of engineering, materials science, chemistry, and environmental science - Research Institutes: National laboratories, industry-funded research centers   Non-Governmental Organizations (NGOs)   - Environmental Groups: Greenpeace, Sierra Club, World Wildlife Fund - Consumer Advocacy Groups: Consumer Reports, National Safety Council   Other Potential Stakeholders   - Investors: Venture capital firms, private equity firms - Supply Chain Consultants: Logistics companies, supply chain management experts - Policy Makers: Government officials, legislators |

### Consolidated comparison of AI-tools output

✓ = Pre-consolidated stakeholder group was covered by the AI tool

() = Term which the AI-tool used for the pre-consolidated stakeholder group

X = Pre-consolidated stakeholder group was not covered by the AI tool

| Pre-consolidated stakeholder group | ChatGPT | Microsoft Co-Pilot | Perplexity AI | Google Gemini |
| --- | --- | --- | --- | --- |
| EV manufacturing | ✓ (Automobile Manufacturers (OEMs)) | ✓ (Automotive Manufacturers) | ✓ (Automotive Manufacturers) | ✓ (Automotive Manufacturers) |
| Battery manufacturing (pack) | ✓ (Battery Pack Assemblers) | ✓ (Battery Manufacturers) | ✓ (Battery Manufacturers) | ✓ (Battery Manufacturers) |
| Battery manufacturing (cell) | ✓ (Battery Cell Manufacturers) | ✓ (Battery Manufacturers) | ✓ (Battery Manufacturers) | ✓ (Battery Manufacturers) |
| Suppliers in the battery industry | ✓ (Raw Material Suppliers) | X | X | ✓ (Raw Material Suppliers) |
| Battery recycling | ✓ (Battery Recycling Companies) | ✓ (Recycling Companies) | ✓ (Battery Recycling Firms) | ✓ (Recycling Companies) |
| Policy maker | ✓ (Government Agencies & Regulators) | ✓ (Government Agencies) | ✓ (Policy Makers) | ✓ (Government Agencies; Policy Makers) |
| Investors | ✓ (Venture Capital & Private Equity Firms; Green Investment Funds) | ✓ (Investors and Venture Capitalists) | ✓ (Venture Capitalists; Banks and Financial Institutions) | X |
| Research | ✓ (Academic Research Groups) | ✓ (Research Institutions) | ✓ (Academic Researchers; Technical Research Organizations) | ✓ (Universities; Research Institutes) |
| Sustainability and circular economy | ✓ (Sustainability and Circular Economy Advocates) | ✓ (Environmental Organizations) | ✓ (Environmental NGOs) | ✓ (Environmental Groups) |
| Disassembly (technology) | ✓ (Disassembly Tool Manufacturers) | ✓ (Technology Providers) | ✓ (Automation Technology Companies) | ✓ (Technology Providers) |
| Logistics | ✓ (Logistics and Transportation Companies) | ✓ (Logistics Providers) | X | X |
| Reuse or repurpose | ✓ (Second-Life-Battery Companies) | X | X | X |
| Safety, hazard and risk management | ✓ (Occupational Safety and Health Bodies; Fire and Hazard Control Specialists) | ✓ (Safety and Compliance Experts) | X | X |
| Waste management |  | x (Waste Management Companies) | x (Waste Management Services) |  |
| Associations |  | x (Industry Associations) | x (Automotive Industry Associations; Battery Industry Associations) |  |
| Consumer watchdog organizations | x (Consumer Watchdog Organizations) |  | x (Consumer Rights Groups) | x (Consumer Advocacy Groups) |

### Results from stakeholder evaluation workshop according to the stakeholder salience model from Mitchell et al. [34]

| Pre-consolidated stakeholder group from AI-tool or workshop | Power | Legitimacy | Urgency | Classification (according to Mitchell et al. [34]) |
| --- | --- | --- | --- | --- |
| EV manufacturing | ✓ |  | ✓ | dangerous Stakeholder |
| Battery manufacturing (pack) | ✓ |  | ✓ | dangerous Stakeholder |
| Battery manufacturing (cell) |  |  |  | Non-relevant Stakeholder |
| Suppliers in the battery industry |  |  |  | Non-relevant Stakeholder |
| Battery recycling | ✓ | ✓ | ✓ | definitive Stakeholder |
| Policy maker | ✓ | ✓ |  | dominant Stakeholder |
| Investors | ✓ |  |  | dormant Stakeholder |
| Research |  | ✓ |  | discretionary Stakeholder |
| Sustainability and circular economy |  | ✓ |  | discretionary Stakeholder |
| Disassembly (technology) | ✓ | ✓ | ✓ | definitive Stakeholder |
| Logistics | ✓ | ✓ | ✓ | definitive Stakeholder |
| Reuse or repurpose | ✓ | ✓ | ✓ | definitive Stakeholder |
| Safety, hazard and risk management | ✓ | ✓ | ✓ | definitive Stakeholder |
| Waste management |  |  |  | Non-relevant Stakeholder |
| Associations |  | ✓ |  | discretionary Stakeholder |
| Consumer watchdog organizations |  |  |  | Non-relevant Stakeholder |
| Disassembly (operation) | ✓ | ✓ | ✓ | definitive Stakeholder |
| Point of extraction |  |  | ✓ | demanding Stakeholder |
| Remanufacturing or refurbishment | ✓ | ✓ | ✓ | definitive Stakeholder |

## Supplementary material for the criteria identification

### Expert characterization for interviews to identify criteria

Expert level:

- Early Professional: 1+ years of work experience
- Advanced Professional: 3+ years of work experience
- Senior Professional: 5+ years of work experience

| Expert number | Academic qualification | Expert level | Worker type | Stakeholder group(s) (see section “Stakeholder selection”, especially Figure 4) |
| --- | --- | --- | --- | --- |
| 1 | University degree | Early Professional | Management | Disassembly companies (operation); Disassembly Technology Providers; Remanufacturing and refurbishment companies; Reuse or repurpose; Sustainability and Circular Economy Experts |
| 2 | University degree | Senior Professional | Management | Disassembly Technology Providers |
| 3 | University degree | Senior Professional | Senior | Battery Manufacturers (Pack); EV Manufacturer; Investors |
| 4 | University degree | Early Professional | Junior | Research |
| 5 | University degree | Senior Professional | Senior | Battery Recycling Companies |
| 6 | University degree | Senior Professional | Management | Disassembly Technology Providers |
| 7 | Doctorate degree | Senior Professional | Senior | Research |
| 8 | Doctorate degree | Senior Professional | Senior | Battery Manufacturers (Pack); Battery Recycling Companies; Investors |
| 9 | Doctorate degree | Senior Professional | Management | Battery Manufacturers (Pack); EV Manufacturer |
| 10 | Doctorate degree | Senior Professional | C-level | Battery Recycling Companies |
| 11 | University degree | Senior Professional | C-level | Battery Recycling Companies; Disassembly companies (operation); Logistics Companies; Point of extraction; Remanufacturing and refurbishment companies; Reuse or repurpose |
| 12 | Doctorate degree | Senior Professional | Senior | Research; Sustainability and Circular Economy Experts |
| 13 | University degree | Senior Professional | Senior | Sustainability and Circular Economy Experts |
| 14 | University degree | Advanced Professional | Management | Research |
| 15 | University degree | Senior Professional | Senior | Disassembly Technology Providers |
| 16 | University degree | Early Professional | Junior | Research |
| 17 | University degree | Senior Professional | Senior | Research |
| 18 | Doctorate degree | Senior Professional | Management | Research |
| 19 | Doctorate degree | Senior Professional | Management | Disassembly Technology Providers |
| 20 | University degree | Senior Professional | Management | Research |
| 21 | University degree | Advanced Professional | Junior | Disassembly Technology Providers |
| 22 | University degree | Senior Professional | Management | Disassembly Technology Providers |
| 23 | University degree | Advanced Professional | Senior | Battery Recycling Companies; Disassembly companies (operation); Logistics Companies; Remanufacturing and refurbishment companies; Reuse or repurpose |
| 24 | University degree | Advanced Professional | Junior | Battery Recycling Companies; Disassembly companies (operation); Remanufacturing and refurbishment companies; Reuse or repurpose |
| 25 | Doctorate degree | Senior Professional | Senior | Battery Manufacturers (Pack); EV Manufacturer; Investors; Research |
| 26 | University degree | Senior Professional | C-level | Disassembly Technology Providers |
| 27 | University degree | Senior Professional | Senior | Disassembly Technology Providers |
| 28 | University degree | Senior Professional | Senior | Disassembly Technology Providers |
| 29 | University degree | Senior Professional | Management | Battery Manufacturers (Pack) |
| 30 | Doctorate degree | Senior Professional | Professor | Research |
| 31 | University degree | Advanced Professional | Senior | Research |
| 32 | University degree | Early Professional | Management | Research |
| 33 | University degree | Early Professional | Junior | Research |
| 34 | University degree | Senior Professional | C-level | Battery Recycling Companies; Disassembly companies (operation) |
| 35 | Doctorate degree | Senior Professional | Management | Sustainability and Circular Economy Experts |
| 36 | Doctorate degree | Senior Professional | Management | Disassembly Technology Providers |
| 37 | University degree | Senior Professional | Management | Disassembly Technology Providers |
| 38 | University degree | Advanced Professional | Management | Battery Recycling Companies; Disassembly companies (operation); Remanufacturing and refurbishment companies; Reuse or repurpose |
| 39 | Doctorate degree | Senior Professional | Senior | Associations; Safety, Hazard and Risk Management; Sustainability and Circular Economy Experts |
| 40 | University degree | Senior Professional | Management | Research |
| 41 | University degree | Senior Professional | Senior | Battery Recycling Companies; Disassembly companies (operation) |
| 42 | Doctorate degree | Senior Professional | Professor | Battery Manufacturers (Pack); EV Manufacturer; Research |
| 43 | Doctorate degree | Senior Professional | Senior | Disassembly Technology Providers |
| 44 | Doctorate degree | Senior Professional | Senior | Disassembly Technology Providers |
| 45 | University degree | Senior Professional | Senior | Sustainability and Circular Economy Experts |
| 46 | Doctorate degree | Senior Professional | Management | Research |
| 47 | University degree | Senior Professional | Professor | Research |
| 48 | University degree | Senior Professional | Senior | Battery Recycling Companies; Disassembly companies (operation); Safety, Hazard and Risk Management |
| 49 | University degree | Senior Professional | Senior | Battery Recycling Companies; Disassembly companies (operation) |
| 50 | University degree | Senior Professional | Management | Disassembly companies (operation) |
| 51 | Doctorate degree | Senior Professional | Management | Battery Manufacturers (Pack); Battery Recycling Companies; Disassembly companies (operation); EV Manufacturer; Research |
| 52 | University degree | Advanced Professional | Senior | Associations; Sustainability and Circular Economy Experts |
| 53 | University degree | Senior Professional | Management | Associations |
| 54 | University degree | Senior Professional | Senior | Associations |
| 55 | University degree | Early Professional | Junior | Policy maker; Sustainability and Circular Economy Experts |
| 56 | University degree | Advanced Professional | Senior | Disassembly Technology Providers |
| 57 | University degree | Senior Professional | C-level | Battery Recycling Companies; Remanufacturing and refurbishment companies; Reuse or repurpose |
| 58 | University degree | Senior Professional | C-level | Battery Recycling Companies |
| 59 | University degree | Senior Professional | Senior | Research |
| 60 | University degree | Senior Professional | C-level | Battery Recycling Companies; Disassembly companies (operation); Logistics Companies; Remanufacturing and refurbishment companies; Reuse or repurpose |
| 61 | University degree | Senior Professional | Senior | Associations |
| 62 | University degree | Senior Professional | Management | Associations |
| 63 | University degree | Advanced Professional | Senior | Policy maker; Sustainability and Circular Economy Experts |
| 64 | Doctorate degree | Senior Professional | Management | Policy maker; Sustainability and Circular Economy Experts |
| 65 | other | Senior Professional | C-level | Point of extraction |

## Supplementary material for the results

### Evaluation criteria from the expert interviews

| Criterion | Quantity of experts mentioning this criterion | Experts (ID) mentioning criterion  (for ID details, please see Table C.1) | Included terms and criteria mentioned by the experts (base for consolidation) | Excerpt of references mentioning criterion |
| --- | --- | --- | --- | --- |
| Adaptability | 37 | 5; 6; 7; 10; 11; 14; 21; 22; 23; 24; 25; 26; 29; 30; 31; 32; 35; 36; 40; 44; 46; 47; 48; 49; 50; 51; 52; 53; 54; 56; 57; 58; 59; 60; 63; 64; 65 | Ability to handle non-standard material; Agility of machinery; Learning ability for upcoming variants; Transformability; Universality | [35]; [36]; [37] |
| Flexibility | 62 | 1; 2; 3; 4; 5; 6; 7; 8; 9; 10; 12; 13; 14; 16; 17; 18; 19; 20; 21; 22; 23; 24; 25; 26; 27; 29; 30; 31; 32; 33; 34; 35; 36; 37; 38; 39; 40; 41; 42; 43; 44; 45; 46; 47; 48; 49; 50; 51; 52; 53; 54; 55; 56; 57; 58; 59; 60; 61; 62; 63; 64; 65 | Ability to handle non-standard material; Ability to handle variability; Ability to handle variance; Ability to handle variety; Ability to reduce sorting effort by handling several variants; Accessibility of different components; Agility of machinery; Applicability to various pack designs; Flexibility in the disassembly of different components; Universality | [38]; [12]; [37] |
| Automatability | 60 | 1; 4; 5; 6; 7; 8; 9; 10; 12; 13; 14; 16; 17; 18; 19; 20; 21; 22; 23; 24; 25; 26; 27; 28; 29; 30; 31; 32; 33; 34; 35; 36; 37; 38; 40; 41; 42; 43; 44; 45; 46; 47; 48; 49; 50; 51; 52; 53; 54; 55; 56; 57; 58; 59; 60; 61; 62; 63; 64; 65 | Automation | [3]; [39]; [40] |
| Capex (Capital expenditure) | 62 | 1; 4; 5; 6; 7; 8; 9; 10; 11; 12; 13; 14; 16; 17; 18; 19; 20; 21; 22; 23; 24; 25; 26; 27; 28; 29; 30; 31; 32; 33; 34; 35; 36; 37; 38; 39; 40; 41; 42; 43; 44; 45; 46; 47; 48; 49; 50; 51; 52; 53; 54; 55; 56; 57; 58; 59; 60; 61; 62; 63; 64; 65 | Approvals; Costs; Expenditures for complying with the law; Incentives; Space requirement; Subventions | [36]; [41]; [42] |
| Opex (Operational expenditure) | 60 | 1; 4; 6; 7; 8; 9; 10; 11; 12; 13; 14; 16; 17; 18; 19; 20; 21; 22; 23; 24; 25; 26; 27; 28; 29; 30; 31; 32; 33; 34; 35; 36; 37; 38; 40; 41; 42; 43; 44; 45; 46; 47; 48; 49; 50; 51; 52; 53; 54; 55; 56; 57; 58; 59; 60; 61; 62; 63; 64; 65 | Costs; Disposal costs of production-related materials; Downtime; Energy efficiency; Labor; Maintenance; Wear | [43]; [44]; [13]; [8] |
| Digital compatibility | 11 | 3; 5; 19; 28; 36; 44; 53; 54; 55; 59; 60 | Adaptability for Artificial intelligence; Artificial intelligence compatibility; Compatibility with battery passport; Documentability; Integration of sensors or human recognition; Traceability | [45]; [46]; [12] |
| Environmental impact | 27 | 7; 10; 19; 23; 25; 26; 30; 33; 35; 37; 38; 39; 40; 41; 43; 44; 45; 48; 49; 50; 51; 54; 55; 57; 58; 61; 65 | Carbon footprint; Disposal of production-related materials; Emission; Energy efficiency; Global warming potential; Environmental Pollution; Sustainability | [37]; [12]; [47] |
| Integratability | 40 | 2; 4; 5; 13; 14; 16; 18; 19; 21; 22; 23; 25; 26; 27; 28; 29; 30; 31; 32; 34; 36; 37; 38; 40; 41; 42; 44; 46; 47; 49; 50; 51; 52; 54; 55; 56; 57; 58; 60; 62 | Integratability for measurement equipment; Integratability into line systems; Integratability into recycling environment; Integratability of quality assurance methods; Media supply; Simplification | Studies only point out that there are no fully automated lines, but they are needed: [38]; [48]; [49] |
| Process robustness and reliability | 45 | 3; 4; 7; 10; 11; 12; 13; 14; 19; 21; 22; 23; 24; 25; 26; 27; 28; 29; 30; 34; 35; 36; 37; 38; 40; 41; 42; 43; 44; 45; 46; 47; 48; 49; 52; 53; 54; 55; 56; 57; 58; 59; 60; 61; 65 | Applicability for multiple materials; Handling of irreversible joined connections; Impact of contamination on the process stability and reliability and robustness; Process reliability; Process repeatability; Process robustness; Process stability | [35]; [24]; [48] |
| Productivity | 55 | 1; 2; 3; 4; 5; 6; 7; 8; 9; 10; 11; 12; 13; 14; 16; 17; 18; 19; 20; 21; 22; 23; 25; 26; 27; 28; 29; 30; 31; 34; 35; 36; 37; 38; 40; 42; 44; 46; 47; 48; 49; 50; 51; 52; 53; 54; 55; 56; 57; 58; 59; 60; 63; 64; 65 | Cycle time; Number of individual disassembly steps; Process complexity; Process simplicity; Process time; Setup time; Speed | References towards time-consuming and complex processes: [50]; [49] |
| Purity of outfeed | 47 | 6; 11; 12; 13; 17; 18; 19; 20; 24; 25; 26; 27; 28; 30; 31; 32; 33; 34; 35; 36; 38; 39; 40; 41; 42; 43; 44; 45; 46; 48; 49; 50; 51; 52; 53; 54; 55; 56; 57; 58; 59; 60; 61; 62; 63; 64; 65 | Compatibility with downstream processes; Depth of disassembly; Quality of outfeed materials; Separability of materials after disassembly | [2]; [51]; [52] |
| Qualification and availability of personnel | 60 | 2; 4; 5; 6; 7; 8; 9; 10; 11; 12; 13; 14; 16; 17; 18; 19; 20; 21; 22; 23; 24; 25; 26; 27; 28; 29; 30; 31; 32; 33; 34; 35; 37; 38; 40; 41; 42; 43; 44; 45; 46; 47; 48; 49; 50; 51; 52; 53; 54; 55; 56; 57; 58; 59; 60; 61; 62; 63; 64; 65 | Expertise in personnel; Effort to qualify personnel | [35]; [42]; [53] |
| Repurposability | 52 | 2; 3; 4; 5; 7; 8; 10; 12; 13; 14; 16; 17; 18; 19; 20; 21; 22; 23; 24; 25; 26; 27; 29; 30; 31; 32; 33; 35; 37; 38; 40; 41; 42; 43; 44; 46; 47; 50; 51; 52; 53; 54; 55; 56; 57; 58; 59; 60; 61; 63; 64; 65 | Degree of destruction; Energy input into the battery cells; Precision of separation operations; Reusability; Repairability | [39]; [13]; [8] |
| Safety | 60 | 2; 3; 4; 5; 6; 7; 8; 9; 10; 11; 13; 14; 16; 17; 18; 19; 20; 21; 22; 23; 24; 25; 26; 27; 28; 29; 30; 31; 32; 33; 34; 35; 36; 37; 38; 39; 40; 41; 42; 43; 44; 46; 47; 48; 49; 50; 51; 52; 53; 54; 55; 56; 57; 58; 59; 60; 62; 63; 64; 65 | Ability to perform disassembly with non-deep-discharged batteries; Energy input (cell); Heat dissipation of disassembly process; Machinery safety; Personnel safety; Precision; Risk of accidents | [53]; [48]; [54] |
| Scalability | 50 | 4; 5; 6; 13; 14; 18; 19; 21; 22; 23; 24; 25; 26; 27; 28; 29; 30; 31; 33; 34; 35; 36; 37; 38; 40; 41; 42; 43; 44; 45; 46; 47; 48; 49; 50; 51; 52; 53; 54; 55; 56; 57; 58; 59; 60; 61; 62; 63; 64; 65 | Modularity; Parallelization of disassembly steps; Relocatability; Standardizability | [35]; [38]; [9] |
| Technology readiness | 32 | 6; 11; 14; 21; 22; 23; 24; 25; 26; 29; 30; 35; 36; 40; 43; 44; 46; 47; 48; 49; 50; 51; 52; 53; 54; 56; 57; 58; 60; 63; 64; 65 | Accessibility of technology; Technology acceptance (bias) | References mention that there are no (automated) plants ready yet, and current technologies are in an early stage: [38]; [12]; [49] |

## Expert ratings from the delphi panel

The expert ratings are documented in an Excel file attached to the article.

(see Supplementary Material 2)

## Aggregated results from the delphi panel

### Results of criteria weighting process

| Criterion | Mean $f_{Ec1_{r_{2}}}$ | Mean $f_{Ec2_{r_{2}}}$ | Mean $f_{Ec3_{r_{2}}}$ | Mean $CoG_{i_{Ec}(r_{2})}$ |
| --- | --- | --- | --- | --- |
| Adaptability | 0.6692 | 0.8538 | 0.9692 | 0.8308 |
| Flexibility | 0.7000 | 0.8692 | 0.9692 | 0.8462 |
| Automatability | 0.5923 | 0.7923 | 0.9308 | 0.7718 |
| Capex | 0.6692 | 0.8615 | 0.9769 | 0.8359 |
| Opex | 0.6846 | 0.8692 | 0.9769 | 0.8436 |
| Digital compatibility | 0.4538 | 0.6538 | 0.8231 | 0.6436 |
| Environmental impact | 0.5154 | 0.7000 | 0.8692 | 0.6949 |
| Integratability | 0.4538 | 0.6538 | 0.8538 | 0.6538 |
| Process robustness and reliability | 0.7923 | 0.9462 | 1.0000 | 0.9128 |
| Productivity | 0.7308 | 0.9154 | 1.0000 | 0.8821 |
| Purity of outfeed | 0.6538 | 0.8308 | 0.9538 | 0.8128 |
| Qualification and availability of personnel | 0.4231 | 0.6231 | 0.8154 | 0.6205 |
| Repurposability | 0.2231 | 0.4077 | 0.6077 | 0.4128 |
| Safety | 0.7923 | 0.9385 | 0.9923 | 0.9077 |
| Scalability | 0.7000 | 0.8769 | 0.9692 | 0.8487 |
| Technology readiness | 0.6846 | 0.8615 | 0.9692 | 0.8385 |

### Results of criteria-based technology evaluation process

| Criterion & Technology | Mean $f_{Ect1_{r_{2}}}$ | Mean $f_{Ect2_{r_{2}}}$ | Mean $f_{Ect3_{r_{2}}}$ | Mean $CoG_{i_{Ect}(r_{2})}$ |
| --- | --- | --- | --- | --- |
| Adaptability & Laser | 0.6231 | 0.8154 | 0.9462 | 0.7949 |
| Adaptability & Shear / Knife | 0.4077 | 0.6077 | 0.8077 | 0.6077 |
| Adaptability & Milling | 0.4462 | 0.6308 | 0.8077 | 0.6282 |
| Adaptability & Rotary cutting tool | 0.4462 | 0.6308 | 0.8154 | 0.6308 |
| Adaptability & Water jet | 0.4462 | 0.6308 | 0.8000 | 0.6256 |
| Adaptability & Robotic unscrewing | 0.3000 | 0.4615 | 0.6385 | 0.4667 |
| Adaptability & Toolless manual removal | 0.8077 | 0.9385 | 0.9846 | 0.9103 |
| Adaptability & Shredding | 0.8385 | 0.9692 | 1.0000 | 0.9359 |
| Flexibility & Laser | 0.6385 | 0.8308 | 0.9615 | 0.8103 |
| Flexibility & Shear / Knife | 0.3923 | 0.5923 | 0.7923 | 0.5923 |
| Flexibility & Milling | 0.4308 | 0.6154 | 0.7923 | 0.6128 |
| Flexibility & Rotary cutting tool | 0.4308 | 0.6154 | 0.8000 | 0.6154 |
| Flexibility & Water jet | 0.4615 | 0.6462 | 0.8154 | 0.6410 |
| Flexibility & Robotic unscrewing | 0.3308 | 0.4846 | 0.6538 | 0.4897 |
| Flexibility & Toolless manual removal | 0.8077 | 0.9385 | 0.9846 | 0.9103 |
| Flexibility & Shredding | 0.8385 | 0.9692 | 1.0000 | 0.9359 |
| Automatability & Laser | 0.7923 | 0.9462 | 1.0000 | 0.9128 |
| Automatability & Shear / Knife | 0.6077 | 0.8077 | 0.9385 | 0.7846 |
| Automatability & Milling | 0.6000 | 0.7923 | 0.9231 | 0.7718 |
| Automatability & Rotary cutting tool | 0.4615 | 0.6462 | 0.7923 | 0.6333 |
| Automatability & Water jet | 0.6308 | 0.8154 | 0.9231 | 0.7897 |
| Automatability & Robotic unscrewing | 0.3538 | 0.5385 | 0.7154 | 0.5359 |
| Automatability & Toolless manual removal | 0.0000 | 0.0000 | 0.1000 | 0.0333 |
| Automatability & Shredding | 0.8385 | 0.9692 | 1.0000 | 0.9359 |
| Capex & Laser | 0.0462 | 0.1769 | 0.3769 | 0.2000 |
| Capex & Shear / Knife | 0.5308 | 0.7308 | 0.9077 | 0.7231 |
| Capex & Milling | 0.3462 | 0.5462 | 0.7462 | 0.5462 |
| Capex & Rotary cutting tool | 0.4231 | 0.6231 | 0.8154 | 0.6205 |
| Capex & Water jet | 0.2077 | 0.3923 | 0.5923 | 0.3974 |
| Capex & Robotic unscrewing | 0.2462 | 0.4308 | 0.6231 | 0.4333 |
| Capex & Toolless manual removal | 0.8385 | 0.9615 | 0.9923 | 0.9308 |
| Capex & Shredding | 0.3385 | 0.5154 | 0.7077 | 0.5205 |
| Opex & Laser | 0.6231 | 0.8154 | 0.9462 | 0.7949 |
| Opex & Shear / Knife | 0.4231 | 0.6231 | 0.8154 | 0.6205 |
| Opex & Milling | 0.3769 | 0.5769 | 0.7769 | 0.5769 |
| Opex & Rotary cutting tool | 0.4231 | 0.6231 | 0.8231 | 0.6231 |
| Opex & Water jet | 0.1923 | 0.3769 | 0.5769 | 0.3821 |
| Opex & Robotic unscrewing | 0.5000 | 0.7000 | 0.8769 | 0.6923 |
| Opex & Toolless manual removal | 0.0308 | 0.0923 | 0.2385 | 0.1205 |
| Opex & Shredding | 0.3769 | 0.5769 | 0.7692 | 0.5744 |
| Digital compatibility & Laser | 0.7923 | 0.9462 | 1.0000 | 0.9128 |
| Digital compatibility & Shear / Knife | 0.5923 | 0.7923 | 0.9385 | 0.7744 |
| Digital compatibility & Milling | 0.7000 | 0.8846 | 0.9846 | 0.8564 |
| Digital compatibility & Rotary cutting tool | 0.5462 | 0.7462 | 0.9000 | 0.7308 |
| Digital compatibility & Water jet | 0.6692 | 0.8692 | 0.9846 | 0.8410 |
| Digital compatibility & Robotic unscrewing | 0.6846 | 0.8846 | 0.9923 | 0.8538 |
| Digital compatibility & Toolless manual removal | 0.0308 | 0.1308 | 0.3154 | 0.1590 |
| Digital compatibility & Shredding | 0.3923 | 0.5923 | 0.7846 | 0.5897 |
| Environmental impact & Laser | 0.5462 | 0.7385 | 0.8923 | 0.7256 |
| Environmental impact & Shear / Knife | 0.5615 | 0.7615 | 0.9077 | 0.7436 |
| Environmental impact & Milling | 0.4846 | 0.6846 | 0.8462 | 0.6718 |
| Environmental impact & Rotary cutting tool | 0.4538 | 0.6538 | 0.8308 | 0.6462 |
| Environmental impact & Water jet | 0.0923 | 0.2462 | 0.4385 | 0.2590 |
| Environmental impact & Robotic unscrewing | 0.6692 | 0.8538 | 0.9615 | 0.8282 |
| Environmental impact & Toolless manual removal | 0.7923 | 0.9308 | 0.9769 | 0.9000 |
| Environmental impact & Shredding | 0.1923 | 0.3769 | 0.5769 | 0.3821 |
| Integratability & Laser | 0.7462 | 0.9154 | 0.9923 | 0.8846 |
| Integratability & Shear / Knife | 0.5462 | 0.7462 | 0.9077 | 0.7333 |
| Integratability & Milling | 0.6077 | 0.8077 | 0.9385 | 0.7846 |
| Integratability & Rotary cutting tool | 0.5308 | 0.7308 | 0.9000 | 0.7205 |
| Integratability & Water jet | 0.3692 | 0.5615 | 0.7462 | 0.5590 |
| Integratability & Robotic unscrewing | 0.4846 | 0.6846 | 0.8692 | 0.6795 |
| Integratability & Toolless manual removal | 0.4154 | 0.6077 | 0.7692 | 0.5974 |
| Integratability & Shredding | 0.6692 | 0.8538 | 0.9692 | 0.8308 |
| Process robustness and reliability & Laser | 0.6385 | 0.8385 | 0.9692 | 0.8154 |
| Process robustness and reliability & Shear / Knife | 0.4231 | 0.6231 | 0.8231 | 0.6231 |
| Process robustness and reliability & Milling | 0.4154 | 0.6077 | 0.8000 | 0.6077 |
| Process robustness and reliability & Rotary cutting tool | 0.4000 | 0.5923 | 0.7923 | 0.5949 |
| Process robustness and reliability & Water jet | 0.5077 | 0.7000 | 0.8769 | 0.6949 |
| Process robustness and reliability & Robotic unscrewing | 0.2000 | 0.3615 | 0.5538 | 0.3718 |
| Process robustness and reliability & Toolless manual removal | 0.4462 | 0.6385 | 0.8000 | 0.6282 |
| Process robustness and reliability & Shredding | 0.6385 | 0.8231 | 0.9385 | 0.8000 |
| Productivity & Laser | 0.7154 | 0.8846 | 0.9692 | 0.8564 |
| Productivity & Shear / Knife | 0.3462 | 0.5462 | 0.7462 | 0.5462 |
| Productivity & Milling | 0.3846 | 0.5769 | 0.7615 | 0.5744 |
| Productivity & Rotary cutting tool | 0.3385 | 0.5308 | 0.7308 | 0.5333 |
| Productivity & Water jet | 0.3692 | 0.5615 | 0.7462 | 0.5590 |
| Productivity & Robotic unscrewing | 0.1231 | 0.3000 | 0.5000 | 0.3077 |
| Productivity & Toolless manual removal | 0.0615 | 0.1769 | 0.3615 | 0.2000 |
| Productivity & Shredding | 0.8385 | 0.9692 | 1.0000 | 0.9359 |
| Purity of outfeed & Laser | 0.6538 | 0.8462 | 0.9692 | 0.8231 |
| Purity of outfeed & Shear / Knife | 0.5923 | 0.7923 | 0.9462 | 0.7769 |
| Purity of outfeed & Milling | 0.4692 | 0.6692 | 0.8462 | 0.6615 |
| Purity of outfeed & Rotary cutting tool | 0.5154 | 0.7154 | 0.8923 | 0.7077 |
| Purity of outfeed & Water jet | 0.2615 | 0.4385 | 0.6231 | 0.4410 |
| Purity of outfeed & Robotic unscrewing | 0.8077 | 0.9462 | 0.9923 | 0.9154 |
| Purity of outfeed & Toolless manual removal | 0.8077 | 0.9538 | 1.0000 | 0.9205 |
| Purity of outfeed & Shredding | 0.0077 | 0.0385 | 0.1615 | 0.0692 |
| Qualification and availability of personnel & Laser | 0.4154 | 0.6077 | 0.7692 | 0.5974 |
| Qualification and availability of personnel & Shear / Knife | 0.4538 | 0.6538 | 0.8385 | 0.6487 |
| Qualification and availability of personnel & Milling | 0.4231 | 0.6231 | 0.8000 | 0.6154 |
| Qualification and availability of personnel & Rotary cutting tool | 0.4385 | 0.6385 | 0.8231 | 0.6333 |
| Qualification and availability of personnel & Water jet | 0.4692 | 0.6692 | 0.8308 | 0.6564 |
| Qualification and availability of personnel & Robotic unscrewing | 0.2615 | 0.4462 | 0.6231 | 0.4436 |
| Qualification and availability of personnel & Toolless manual removal | 0.3462 | 0.5308 | 0.7154 | 0.5308 |
| Qualification and availability of personnel & Shredding | 0.6077 | 0.8077 | 0.9462 | 0.7872 |
| Repurposability & Laser | 0.4231 | 0.6231 | 0.8231 | 0.6231 |
| Repurposability & Shear / Knife | 0.4385 | 0.6385 | 0.8385 | 0.6385 |
| Repurposability & Milling | 0.3769 | 0.5769 | 0.7769 | 0.5769 |
| Repurposability & Rotary cutting tool | 0.4231 | 0.6231 | 0.8231 | 0.6231 |
| Repurposability & Water jet | 0.0538 | 0.1923 | 0.3923 | 0.2128 |
| Repurposability & Robotic unscrewing | 0.7769 | 0.9385 | 1.0000 | 0.9051 |
| Repurposability & Toolless manual removal | 0.7769 | 0.9385 | 1.0000 | 0.9051 |
| Repurposability & Shredding | 0.0000 | 0.0000 | 0.1000 | 0.0333 |
| Safety & Laser | 0.5615 | 0.7615 | 0.9154 | 0.7462 |
| Safety & Shear / Knife | 0.4538 | 0.6538 | 0.8538 | 0.6538 |
| Safety & Milling | 0.4538 | 0.6538 | 0.8538 | 0.6538 |
| Safety & Rotary cutting tool | 0.4385 | 0.6385 | 0.8385 | 0.6385 |
| Safety & Water jet | 0.2769 | 0.4692 | 0.6615 | 0.4692 |
| Safety & Robotic unscrewing | 0.6538 | 0.8538 | 0.9769 | 0.8282 |
| Safety & Toolless manual removal | 0.2000 | 0.3692 | 0.5615 | 0.3769 |
| Safety & Shredding | 0.5615 | 0.7615 | 0.9077 | 0.7436 |
| Scalability & Laser | 0.6692 | 0.8692 | 0.9769 | 0.8385 |
| Scalability & Shear / Knife | 0.5923 | 0.7923 | 0.9231 | 0.7692 |
| Scalability & Milling | 0.6077 | 0.8077 | 0.9308 | 0.7821 |
| Scalability & Rotary cutting tool | 0.5923 | 0.7923 | 0.9154 | 0.7667 |
| Scalability & Water jet | 0.5154 | 0.7154 | 0.8692 | 0.7000 |
| Scalability & Robotic unscrewing | 0.5000 | 0.7000 | 0.8538 | 0.6846 |
| Scalability & Toolless manual removal | 0.0538 | 0.1769 | 0.3615 | 0.1974 |
| Scalability & Shredding | 0.7154 | 0.8846 | 0.9769 | 0.8590 |
| Technology readiness & Laser | 0.5615 | 0.7538 | 0.9000 | 0.7385 |
| Technology readiness & Shear / Knife | 0.4846 | 0.6846 | 0.8615 | 0.6769 |
| Technology readiness & Milling | 0.4692 | 0.6692 | 0.8538 | 0.6641 |
| Technology readiness & Rotary cutting tool | 0.4692 | 0.6692 | 0.8462 | 0.6615 |
| Technology readiness & Water jet | 0.3462 | 0.5462 | 0.7385 | 0.5436 |
| Technology readiness & Robotic unscrewing | 0.2385 | 0.4231 | 0.6231 | 0.4282 |
| Technology readiness & Toolless manual removal | 0.7769 | 0.9308 | 0.9923 | 0.9000 |
| Technology readiness & Shredding | 0.7462 | 0.9231 | 1.0000 | 0.8897 |

### Change metrics between round 1 and 2 for criteria weighting

| Investigated subject | Expert ID | | | | | | | | | | | | | Mean |
| --- | --- | --- | --- | --- | --- | --- | --- | --- | --- | --- | --- | --- | --- | --- |
|  | 1 | 2 | 3 | 4 | 5 | 6 | 7 | 8 | 9 | 10 | 11 | 12 | 13 |  |
| Number (percentage from total number of items) of to be reevaluated items | 5 (31.25%) | 4  (25%) | 5 (31.25%) | 6 (37.5%) | 5 (31.25%) | 5 (31.25%) | 3 (18.75%) | 4  (25%) | 4  (25%) | 4  (25%) | 3 (18.75%) | 4  (25%) | 4  (25%) | 4.31 (26.92%) |
| Number (percentage from total number of items) of actually changed items | 4  (25%) | 3 (18.75%) | 0  (0%) | 4  (25%) | 2 (12.5%) | 2 (12.5%) | 3 (18.75%) | 3 (18.75%) | 3 (18.75%) | 3 (18.75%) | 1 (6.25%) | 3 (18.75%) | 2 (12.5%) | 2.54 (15.87%) |
| Number (percentage from changed items) of changes with one stage | 3  (75%) | 1 (33.33%) | 0  (0%) | 3  (75%) | 0  (0%) | 2  (100%) | 3  (100%) | 3  (100%) | 1 (33.33%) | 2 (66.67%) | 1  (100%) | 1 (33.33%) | 1  (50%) | 1.62 (63.64%) |
| Number (percentage from changed items) of changes with two stages | 1  (25%) | 2 (66.67%) | 0  (0%) | 1  (25%) | 1  (50%) | 0  (0%) | 0  (0%) | 0  (0%) | 2 (66.67%) | 1 (33.33%) | 0  (0%) | 0  (0%) | 0  (0%) | 0.62 (24.24%) |
| Number (percentage from changed items) of changes with three stages | 0  (0%) | 0  (0%) | 0  (0%) | 0  (0%) | 1  (50%) | 0  (0%) | 0  (0%) | 0  (0%) | 0  (0%) | 0  (0%) | 0  (0%) | 2 (66.67%) | 1  (50%) | 0.31 (12.12%) |
| Number (percentage from changed items) of changes with four stages | 0  (0%) | 0  (0%) | 0  (0%) | 0  (0%) | 0  (0%) | 0  (0%) | 0  (0%) | 0  (0%) | 0  (0%) | 0  (0%) | 0  (0%) | 0  (0%) | 0  (0%) | 0  (0%) |
| Number (percentage from changed items) of changes with five stages | 0  (0%) | 0  (0%) | 0  (0%) | 0  (0%) | 0  (0%) | 0  (0%) | 0  (0%) | 0  (0%) | 0  (0%) | 0  (0%) | 0  (0%) | 0  (0%) | 0  (0%) | 0  (0%) |
| Number (percentage from changed items) of changes with six stages | 0  (0%) | 0  (0%) | 0  (0%) | 0  (0%) | 0  (0%) | 0  (0%) | 0  (0%) | 0  (0%) | 0  (0%) | 0  (0%) | 0  (0%) | 0  (0%) | 0  (0%) | 0  (0%) |
| Number (percentage from actually changed items) of changed tendency (incl. neutral) | 4  (100%) | 3  (100%) | 0  (0%) | 1  (25%) | 2  (100%) | 0  (0%) | 1 (33.33%) | 1 (33.33%) | 1 (33.33%) | 3  (100%) | 0  (0%) | 2 (66.67%) | 2  (100%) | 1.54 (53.21%) |
| Number (percentage from actually changed items) of changed tendency (excl. neutral) | 1  (25%) | 2 (66.67%) | 0  (0%) | 1  (25%) | 2  (100%) | 0  (0%) | 0  (0%) | 0  (0%) | 0  (0%) | 1 (33.33%) | 0  (0%) | 2 (66.67%) | 1  (50%) | 0.77 (28.21%) |

### Change metrics between round 1 and 2 for criteria-based technology evaluation

| Investigated subject | Expert ID | | | | | | | | | | | | | Mean |
| --- | --- | --- | --- | --- | --- | --- | --- | --- | --- | --- | --- | --- | --- | --- |
|  | 1 | 2 | 3 | 4 | 5 | 6 | 7 | 8 | 9 | 10 | 11 | 12 | 13 |  |
| Number (percentage from total number of items) of to be reevaluated items | 52 (40.63%) | 73 (57.03%) | 52 (40.63%) | 58 (45.31%) | 53 (41.41%) | 45 (35.16%) | 53 (41.41%) | 26 (20.31%) | 63 (49.22%) | 33 (25.78%) | 50 (39.06%) | 65 (50.78%) | 43 (33.59%) | 51.23 (40.02%) |
| Number (percentage from total number of items) of actually changed items | 33 (25.78%) | 13 (10.16%) | 29 (22.66%) | 39 (30.47%) | 22 (17.19%) | 17 (13.28%) | 33 (25.78%) | 10 (7.81%) | 48 (37.5%) | 14 (10.94%) | 23 (17.97%) | 37 (28.91%) | 31 (24.22%) | 26.85 (20.97%) |
| Number (percentage from changed items) of changes with one stage | 12 (36.36%) | 3 (23.08%) | 15 (51.72%) | 11 (28.21%) | 14 (63.64%) | 11 (64.71%) | 30 (90.91%) | 8  (80%) | 26 (54.17%) | 13 (92.86%) | 23 (100%) | 22 (59.46%) | 15 (48.39%) | 15.62 (58.17%) |
| Number (percentage from changed items) of changes with two stages | 20 (60.61%) | 6 (46.15%) | 11 (37.93%) | 17 (43.59%) | 7 (31.82%) | 4 (23.53%) | 3 (9.09%) | 2  (20%) | 17 (35.42%) | 1 (7.14%) | 0  (0%) | 8 (21.62%) | 7 (22.58%) | 7.92 (29.51%) |
| Number (percentage from changed items) of changes with three stages | 1 (3.03%) | 1 (7.69%) | 2  (6.9%) | 7 (17.95%) | 1 (4.55%) | 2 (11.76%) | 0  (0%) | 0  (0%) | 5 (10.42%) | 0  (0%) | 0  (0%) | 6 (16.22%) | 7 (22.58%) | 2.46 (9.17%) |
| Number (percentage from changed items) of changes with four stages | 0  (0%) | 3 (23.08%) | 1 (3.45%) | 4 (10.26%) | 0  (0%) | 0  (0%) | 0  (0%) | 0  (0%) | 0  (0%) | 0  (0%) | 0  (0%) | 1  (2.7%) | 0  (0%) | 0.69 (2.58%) |
| Number (percentage from changed items) of changes with five stages | 0  (0%) | 0  (0%) | 0  (0%) | 0  (0%) | 0  (0%) | 0  (0%) | 0  (0%) | 0  (0%) | 0  (0%) | 0  (0%) | 0  (0%) | 0  (0%) | 1 (3.23%) | 0.08 (0.29%) |
| Number (percentage from changed items) of changes with six stages | 0  (0%) | 0  (0%) | 0  (0%) | 0  (0%) | 0  (0%) | 0  (0%) | 0  (0%) | 0  (0%) | 0  (0%) | 0  (0%) | 0  (0%) | 0  (0%) | 1 (3.23%) | 0.08 (0.29%) |
| Number (percentage from actually changed items) of changed tendency (incl. neutral) | 25 (75.76%) | 12 (92.31%) | 31 (106.9%) | 34 (87.18%) | 12 (54.55%) | 14 (82.35%) | 7 (21.21%) | 8  (80%) | 30 (62.5%) | 9 (64.29%) | 11 (47.83%) | 27 (72.97%) | 22 (70.97%) | 18.62 (70.68%) |
| Number (percentage from actually changed items) of changed tendency (excl. neutral) | 12 (36.36%) | 7 (53.85%) | 7 (24.14%) | 18 (46.15%) | 2 (9.09%) | 5 (29.41%) | 2 (6.06%) | 0  (0%) | 7 (14.58%) | 0  (0%) | 0  (0%) | 12 (32.43%) | 6 (19.35%) | 6 (20.88%) |

## Supplementary material for the sensitivity analysis

The subsequent tables present the outcomes of the sensitivity analysis in different scenarios.

### Sensitivity analysis for criterion importance variation (+2)

| Varied criterion (+2) | Ranking change | New final score | | | | | | | |
| --- | --- | --- | --- | --- | --- | --- | --- | --- | --- |
|  |  | Laser | Shredding | Shear / Knife | Milling | Rotary cutting tool | Robotic unscrewing | Toolless manual removal | Water jet |
| Adaptability | none | 0.7581 | 0.7037 | 0.6787 | 0.6587 | 0.6466 | 0.5996 | 0.5715 | 0.5567 |
| Flexibility | none | 0.7583 | 0.7034 | 0.6786 | 0.6586 | 0.6465 | 0.6000 | 0.5711 | 0.5568 |
| Automatability | none | 0.7600 | 0.7045 | 0.6810 | 0.6607 | 0.6466 | 0.6001 | 0.5601 | 0.5593 |
| Capex | none | 0.7519 | 0.6993 | 0.6799 | 0.6579 | 0.6465 | 0.5993 | 0.5716 | 0.5543 |
| Opex | none | 0.7581 | 0.6999 | 0.6789 | 0.6582 | 0.6466 | 0.6019 | 0.5634 | 0.5542 |
| Digital compatability | yes Toolless manual removal  (original rank: 7 \| new rank: 8)  and Water jet  (original rank: 8 \| new rank: 7) changed the rank | 0.7609 | 0.6989 | 0.6814 | 0.6631 | 0.6485 | 0.6062 | 0.5594 | 0.5618 |
| Environmental impact | none | 0.7571 | 0.6949 | 0.6808 | 0.6593 | 0.6468 | 0.6055 | 0.5743 | 0.5501 |
| Integratability | none | 0.7607 | 0.7041 | 0.6807 | 0.6619 | 0.6485 | 0.6028 | 0.5684 | 0.5560 |
| Process robustness and reliability | none | 0.7580 | 0.7016 | 0.6792 | 0.6588 | 0.6466 | 0.6001 | 0.5680 | 0.5566 |
| Productivity | none | 0.7584 | 0.7028 | 0.6786 | 0.6585 | 0.6460 | 0.5991 | 0.5653 | 0.5560 |
| Purity of outfeed | none | 0.7585 | 0.6934 | 0.6807 | 0.6591 | 0.6475 | 0.6049 | 0.5721 | 0.5545 |
| Qualification and availablity of personnel | none | 0.7539 | 0.7032 | 0.6788 | 0.6580 | 0.6465 | 0.5973 | 0.5669 | 0.5583 |
| Repurposability | none | 0.7539 | 0.6822 | 0.6783 | 0.6567 | 0.6461 | 0.6097 | 0.5774 | 0.5462 |
| Safety | none | 0.7577 | 0.7014 | 0.6794 | 0.6590 | 0.6468 | 0.6021 | 0.5669 | 0.5555 |
| Scalability | none | 0.7585 | 0.7026 | 0.6803 | 0.6601 | 0.6479 | 0.6018 | 0.5645 | 0.5572 |
| Technology readiness | none | 0.7575 | 0.7031 | 0.6795 | 0.6591 | 0.6469 | 0.5993 | 0.5712 | 0.5558 |

### Sensitivity analysis for criterion importance variation (+1)

| Varied criterion (+1) | Ranking change | New final score | | | | | | | |
| --- | --- | --- | --- | --- | --- | --- | --- | --- | --- |
|  |  | Laser | Shredding | Shear / Knife | Milling | Rotary cutting tool | Robotic unscrewing | Toolless manual removal | Water jet |
| Adaptability | none | 0.7581 | 0.7031 | 0.6789 | 0.6588 | 0.6467 | 0.5999 | 0.5707 | 0.5565 |
| Flexibility | none | 0.7581 | 0.7029 | 0.6789 | 0.6587 | 0.6466 | 0.6002 | 0.5702 | 0.5566 |
| Automatability | none | 0.7594 | 0.7036 | 0.6806 | 0.6602 | 0.6467 | 0.6004 | 0.5622 | 0.5584 |
| Capex | none | 0.7529 | 0.6996 | 0.6799 | 0.6581 | 0.6466 | 0.5996 | 0.5709 | 0.5546 |
| Opex | none | 0.7580 | 0.7001 | 0.6790 | 0.6584 | 0.6466 | 0.6018 | 0.5642 | 0.5546 |
| Digital compatability | none | 0.7597 | 0.6997 | 0.6807 | 0.6616 | 0.6479 | 0.6043 | 0.5626 | 0.5596 |
| Environmental impact | none | 0.7574 | 0.6974 | 0.6803 | 0.6592 | 0.6468 | 0.6038 | 0.5717 | 0.5524 |
| Integratability | none | 0.7595 | 0.7030 | 0.6802 | 0.6608 | 0.6478 | 0.6021 | 0.5682 | 0.5560 |
| Process robustness and reliability | none | 0.7580 | 0.7016 | 0.6792 | 0.6588 | 0.6466 | 0.6001 | 0.5680 | 0.5566 |
| Productivity | none | 0.7584 | 0.7028 | 0.6786 | 0.6585 | 0.6460 | 0.5991 | 0.5653 | 0.5560 |
| Purity of outfeed | none | 0.7583 | 0.6957 | 0.6803 | 0.6591 | 0.6473 | 0.6038 | 0.5708 | 0.5550 |
| Qualification and availablity of personnel | none | 0.7555 | 0.7024 | 0.6791 | 0.6584 | 0.6466 | 0.5988 | 0.5672 | 0.5574 |
| Repurposability | none | 0.7557 | 0.6911 | 0.6789 | 0.6578 | 0.6464 | 0.6056 | 0.5729 | 0.5508 |
| Safety | none | 0.7577 | 0.7013 | 0.6794 | 0.6590 | 0.6468 | 0.6020 | 0.5670 | 0.5556 |
| Scalability | none | 0.7583 | 0.7023 | 0.6801 | 0.6599 | 0.6477 | 0.6017 | 0.5651 | 0.5570 |
| Technology readiness | none | 0.7576 | 0.7026 | 0.6795 | 0.6591 | 0.6469 | 0.5997 | 0.5704 | 0.5559 |

### Sensitivity analysis for criterion importance variation (-1)

| Varied criterion (-1) | Ranking change | New final score | | | | | | | |
| --- | --- | --- | --- | --- | --- | --- | --- | --- | --- |
|  |  | Laser | Shredding | Shear / Knife | Milling | Rotary cutting tool | Robotic unscrewing | Toolless manual removal | Water jet |
| Adaptability | none | 0.7572 | 0.6980 | 0.6805 | 0.6595 | 0.6470 | 0.6029 | 0.5631 | 0.5550 |
| Flexibility | none | 0.7571 | 0.6982 | 0.6806 | 0.6596 | 0.6472 | 0.6025 | 0.5634 | 0.5549 |
| Automatability | none | 0.7555 | 0.6977 | 0.6779 | 0.6574 | 0.6470 | 0.6020 | 0.5756 | 0.5525 |
| Capex | none | 0.7655 | 0.7037 | 0.6789 | 0.6606 | 0.6472 | 0.6034 | 0.5627 | 0.5582 |
| Opex | none | 0.7572 | 0.7029 | 0.6803 | 0.6602 | 0.6471 | 0.5998 | 0.5738 | 0.5583 |
| Digital compatability | none | 0.7554 | 0.7029 | 0.6781 | 0.6561 | 0.6455 | 0.5972 | 0.5740 | 0.5516 |
| Environmental impact | none | 0.7582 | 0.7060 | 0.6785 | 0.6589 | 0.6468 | 0.5976 | 0.5627 | 0.5605 |
| Integratability | none | 0.7557 | 0.6990 | 0.6786 | 0.6570 | 0.6456 | 0.5998 | 0.5673 | 0.5559 |
| Process robustness and reliability | none | 0.7571 | 0.7001 | 0.6801 | 0.6596 | 0.6474 | 0.6036 | 0.5671 | 0.5544 |
| Productivity | none | 0.7565 | 0.6982 | 0.6812 | 0.6601 | 0.6482 | 0.6048 | 0.5725 | 0.5559 |
| Purity of outfeed | none | 0.7569 | 0.7098 | 0.6782 | 0.6590 | 0.6460 | 0.5968 | 0.5629 | 0.5575 |
| Qualification and availablity of personnel | none | 0.7603 | 0.6998 | 0.6800 | 0.6598 | 0.6470 | 0.6036 | 0.5684 | 0.5543 |
| Repurposability | none | 0.7597 | 0.7107 | 0.6801 | 0.6602 | 0.6471 | 0.5967 | 0.5629 | 0.5609 |
| Safety | none | 0.7579 | 0.7007 | 0.6798 | 0.6591 | 0.6469 | 0.5986 | 0.5698 | 0.5569 |
| Scalability | none | 0.7567 | 0.6991 | 0.6783 | 0.6575 | 0.6453 | 0.6000 | 0.5725 | 0.5541 |
| Technology readiness | none | 0.7580 | 0.6987 | 0.6795 | 0.6590 | 0.6466 | 0.6033 | 0.5634 | 0.5561 |

### Sensitivity analysis for criterion importance variation (-2)

| Varied criterion (-2) | Ranking change | New final score | | | | | | | |
| --- | --- | --- | --- | --- | --- | --- | --- | --- | --- |
|  |  | Laser | Shredding | Shear / Knife | Milling | Rotary cutting tool | Robotic unscrewing | Toolless manual removal | Water jet |
| Adaptability | none | 0.7566 | 0.6941 | 0.6817 | 0.6600 | 0.6473 | 0.6051 | 0.5574 | 0.5539 |
| Flexibility | none | 0.7562 | 0.6944 | 0.6820 | 0.6604 | 0.6477 | 0.6043 | 0.5579 | 0.5535 |
| Automatability | none | 0.7529 | 0.6938 | 0.6762 | 0.6555 | 0.6472 | 0.6031 | 0.5845 | 0.5487 |
| Capex | yes Toolless manual removal  (original rank: 7 \| new rank: 8)  and Water jet  (original rank: 8 \| new rank: 7) changed the rank | 0.7748 | 0.7067 | 0.6782 | 0.6625 | 0.6476 | 0.6062 | 0.5567 | 0.5608 |
| Opex | none | 0.7566 | 0.7050 | 0.6813 | 0.6615 | 0.6475 | 0.5983 | 0.5811 | 0.5612 |
| Digital compatability | none | 0.7531 | 0.7045 | 0.6766 | 0.6531 | 0.6443 | 0.5934 | 0.5801 | 0.5474 |
| Environmental impact | yes Toolless manual removal  (original rank: 7 \| new rank: 8)  and Water jet  (original rank: 8 \| new rank: 7) changed the rank | 0.7587 | 0.7111 | 0.6775 | 0.6587 | 0.6468 | 0.5940 | 0.5575 | 0.5652 |
| Integratability | none | 0.7536 | 0.6969 | 0.6777 | 0.6549 | 0.6444 | 0.5985 | 0.5668 | 0.5559 |
| Process robustness and reliability | none | 0.7562 | 0.6985 | 0.6810 | 0.6604 | 0.6482 | 0.6072 | 0.5662 | 0.5523 |
| Productivity | none | 0.7549 | 0.6943 | 0.6834 | 0.6615 | 0.6501 | 0.6096 | 0.5785 | 0.5559 |
| Purity of outfeed | yes Toolless manual removal  (original rank: 7 \| new rank: 8)  and Water jet  (original rank: 8 \| new rank: 7) changed the rank | 0.7558 | 0.7201 | 0.6766 | 0.6590 | 0.6450 | 0.5916 | 0.5572 | 0.5594 |
| Qualification and availablity of personnel | none | 0.7629 | 0.6984 | 0.6805 | 0.6604 | 0.6472 | 0.6061 | 0.5689 | 0.5528 |
| Repurposability | yes Toolless manual removal  (original rank: 7 \| new rank: 8)  and Water jet  (original rank: 8 \| new rank: 7) changed the rank | 0.7611 | 0.7178 | 0.6805 | 0.6611 | 0.6474 | 0.5935 | 0.5594 | 0.5645 |
| Safety | none | 0.7580 | 0.7001 | 0.6802 | 0.6592 | 0.6470 | 0.5951 | 0.5728 | 0.5582 |
| Scalability | none | 0.7554 | 0.6966 | 0.6769 | 0.6555 | 0.6433 | 0.5986 | 0.5784 | 0.5518 |
| Technology readiness | none | 0.7583 | 0.6956 | 0.6796 | 0.6589 | 0.6464 | 0.6061 | 0.5580 | 0.5563 |

### Sensitivity (leave-one-out) analysis for Experts

| Expert left out | Ranking change | New final score | | | | | | | |
| --- | --- | --- | --- | --- | --- | --- | --- | --- | --- |
|  |  | Laser | Shredding | Shear / Knife | Milling | Rotary cutting tool | Robotic unscrewing | Toolless manual removal | Water jet |
| Expert 1 | none | 0.7545 | 0.6993 | 0.6772 | 0.6514 | 0.6511 | 0.6034 | 0.5682 | 0.5519 |
| Expert 2 | none | 0.7565 | 0.6952 | 0.6880 | 0.6815 | 0.6657 | 0.6034 | 0.5735 | 0.5663 |
| Expert 3 | yes Toolless manual removal  (original rank: 7 \| new rank: 8)  and Water jet  (original rank: 8 \| new rank: 7)  changed the rank | 0.7650 | 0.7007 | 0.6788 | 0.6645 | 0.6497 | 0.5961 | 0.5656 | 0.5659 |
| Expert 4 | none | 0.7560 | 0.7000 | 0.6762 | 0.6541 | 0.6445 | 0.6111 | 0.5686 | 0.5509 |
| Expert 5 | none | 0.7601 | 0.7050 | 0.6797 | 0.6501 | 0.6369 | 0.6098 | 0.5734 | 0.5543 |
| Expert 6 | none | 0.7576 | 0.7016 | 0.6803 | 0.6635 | 0.6471 | 0.5965 | 0.5668 | 0.5547 |
| Expert 7 | none | 0.7509 | 0.6998 | 0.6750 | 0.6558 | 0.6412 | 0.5978 | 0.5711 | 0.5503 |
| Expert 8 | none | 0.7582 | 0.7014 | 0.6777 | 0.6577 | 0.6422 | 0.5916 | 0.5681 | 0.5560 |
| Expert 9 | none | 0.7563 | 0.6982 | 0.6848 | 0.6631 | 0.6497 | 0.6112 | 0.5663 | 0.5642 |
| Expert 10 | none | 0.7540 | 0.7023 | 0.6787 | 0.6526 | 0.6439 | 0.5986 | 0.5606 | 0.5540 |
| Expert 11 | none | 0.7637 | 0.7110 | 0.6819 | 0.6597 | 0.6464 | 0.5907 | 0.5664 | 0.5547 |
| Expert 12 | none | 0.7546 | 0.6959 | 0.6748 | 0.6535 | 0.6432 | 0.5964 | 0.5668 | 0.5519 |
| Expert 13 | none | 0.7632 | 0.7048 | 0.6803 | 0.6599 | 0.6467 | 0.6069 | 0.5655 | 0.5523 |

### Sensitivity analysis for defuzzification method variation

| Defuzzification method | Ranking change | New final score | | | | | | | |
| --- | --- | --- | --- | --- | --- | --- | --- | --- | --- |
|  |  | Laser | Shredding | Shear / Knife | Milling | Rotary cutting tool | Robotic unscrewing | Toolless manual removal | Water jet |
| Mean of Maximum | none | 0.7579 | 0.7026 | 0.6792 | 0.6588 | 0.6466 | 0.6000 | 0.5666 | 0.5565 |
| Signed Distance Method | none | 0.7578 | 0.7015 | 0.6794 | 0.6590 | 0.6467 | 0.6008 | 0.5675 | 0.5561 |
| Graded Mean Integration Representative | none | 0.7578 | 0.7019 | 0.6794 | 0.6589 | 0.6467 | 0.6005 | 0.5672 | 0.5562 |

References

1. Rettenmeier, M., Möller, M. & Sauer, A. Disassembly technologies of end-of-life automotive battery packs as the cornerstone for a circular battery value chain: A process-oriented analysis. *Resources, Conservation and Recycling* **209,** 107786. https://doi.org/10.1016/j.resconrec.2024.107786 (2024).

2. Ali, H., Khan, H. A. & Pecht, M. Preprocessing of spent lithium-ion batteries for recycling: Need, methods, and trends. *Renewable and Sustainable Energy Reviews* **168,** 112809. https://doi.org/10.1016/j.rser.2022.112809 (2022).

3. Bhar, M., Ghosh, S., Krishnamurthy, S., Kaliprasad, Y. & Martha, S. K. A review on spent lithium-ion battery recycling: from collection to black mass recovery. *RSC Sustain.* **1,** 1150–1167. https://doi.org/10.1039/D3SU00086A (2023).

4. Wuschke, L., Jäckel, H.-G., Leißner, T. & Peuker, U. A. Crushing of large Li-ion battery cells. *Waste management (New York, N.Y.)* **85,** 317–326. https://doi.org/10.1016/j.wasman.2018.12.042 (2019).

5. Zhao, G. Reuse and Recycling of Lithium‐Ion Power Batteries. https://doi.org/10.1002/9781119321866 (2017).

6. Fleischer, J., Gerlitz, E., Rieβ, S., Coutandin, S. & Hofmann, J. Concepts and Requirements for Flexible Disassembly Systems for Drive Train Components of Electric Vehicles. *Procedia CIRP* **98,** 577–582. https://doi.org/10.1016/j.procir.2021.01.154 (2021).

7. Graner, M. *et al.* Requirements for a Process to Remanufacture EV Battery Packs Down to Cell Level and Necessary Design Modifications. *Advances in Automotive Production Technology – Towards Software-Defined Manufacturing and Resilient Supply Chains (Stuttgart Conference on Automotive Production),* 376–386. https://doi.org/10.1007/978-3-031-27933-1_35 (2022).

8. Rallo, H., Benveniste, G., Gestoso, I. & Amante, B. Economic analysis of the disassembling activities to the reuse of electric vehicles Li-ion batteries. *Resources, Conservation and Recycling* **159,** 104785. https://doi.org/10.1016/j.resconrec.2020.104785 (2020).

9. Weyrich, M. & Natkunaraiah, N. Conception of an automated plant for the disassembly of lithium ion batteries. *The 6th International Conference on Life Cyle Management (LCM) 2013* (2013).

10. Kampker, A., Wessel, S., Fiedler, F. & Maltoni, F. Battery pack remanufacturing process up to cell level with sorting and repurposing of battery cells. *Jnl Remanufactur* **11,** 1–23. https://doi.org/10.1007/s13243-020-00088-6 (2021).

11. Kay, I., Farhad, S., Mahajan, A., Esmaeeli, R. & Hashemi, S. R. Robotic Disassembly of Electric Vehicles’ Battery Modules for Recycling. *Energies* **15,** 4856. https://doi.org/10.3390/en15134856 (2022).

12. Meng, K., Xu, G., Peng, X., Youcef-Toumi, K. & Li, J. Intelligent disassembly of electric-vehicle batteries: a forward-looking overview. *Resources, Conservation and Recycling* **182,** 106207. https://doi.org/10.1016/j.resconrec.2022.106207 (2022).

13. Rosenberg, S. *et al.* Field Study and Multimethod Analysis of an EV Battery System Disassembly. *Energies* **15,** 5324. https://doi.org/10.3390/en15155324 (2022).

14. Korneliussen, J. T. & Zubaidi, O. University of Agder, 2022, https://hdl.handle.net/11250/3070216 Access on 06/19/2025.

15. Pražanová, A., Míka, M. H. & Knap, V. Lithium-ion battery module-to-cell: disassembly and material analysis. *J. Phys.: Conf. Ser.* **2382,** 12002. https://doi.org/10.1088/1742-6596/2382/1/012002 (2022).

16. Schäfer, J., Singer, R., Hofmann, J. & Fleischer, J. Challenges and Solutions of Automated Disassembly and Condition-Based Remanufacturing of Lithium-Ion Battery Modules for a Circular Economy. *Procedia Manufacturing* **43,** 614–619. https://doi.org/10.1016/j.promfg.2020.02.145 (2020).

17. Yin, H., Xiao, J. & Wang, G. Human-Robot Collaboration Re-Manufacturing for Uncertain Disassembly in Retired Battery Recycling. *5th World Conference on Mechanical Engineering and Intelligent Manufacturing (WCMEIM),* 595–598. https://doi.org/10.1109/WCMEIM56910.2022.10021388 (2022).

18. Zhang, S. *et al.* Design and Implementation of a Multifunctional Screw Disassembly Workstation. *Lecture Notes in Computer Science* **14274,** 506–519. https://doi.org/10.1007/978-981-99-6501-4_43 (2023).

19. Hathaway, J. *et al.* Towards reuse and recycling of lithium-ion batteries: tele-robotics for disassembly of electric vehicle batteries. *Frontiers in robotics and AI* **10,** 1179296. https://doi.org/10.3389/frobt.2023.1179296 (2023).

20. Rastegarpanah, A., Hathaway, J. & Stolkin, R. Vision-Guided MPC for Robotic Path Following Using Learned Memory-Augmented Model. *Frontiers in robotics and AI* **8,** 688275. https://doi.org/10.3389/frobt.2021.688275 (2021).

21. Al Assadi, A. *et al.* Machine learning based screw drive state detection for unfastening screw connections. *Journal of Manufacturing Systems* **65,** 19–32. https://doi.org/10.1016/j.jmsy.2022.07.013 (2022).

22. Deng, W. *et al.* Predictive exposure control for vision-based robotic disassembly using deep learning and predictive learning. *Robotics and Computer-Integrated Manufacturing* **85,** 102619. https://doi.org/10.1016/j.rcim.2023.102619 (2024).

23. Li, H. *et al.* An Accurate Activate Screw Detection Method for Automatic Electric Vehicle Battery Disassembly. *Batteries* **9,** 187. https://doi.org/10.3390/batteries9030187 (2023).

24. Chen, W. H., Wegener, K. & Dietrich, F. A robot assistant for unscrewing in hybrid human-robot disassembly. *IEEE International Conference on Robotics and Biomimetics,* 536–541. https://doi.org/10.1109/ROBIO.2014.7090386 (2014).

25. Engelen, B., Marelle, D. de, Kellens, K. & Peeters, J. R. Intuitive teaching approach for robotic disassembly. *Procedia CIRP* **116,** 384–389. https://doi.org/10.1016/j.procir.2023.02.065 (2023).

26. Gadgil, S., Gaikwad, D., Sebastian, B., Dubey, A. & Upadhyay, A. Semi-Autonomous Robotic System for Efficient Recycling of Lithium-Ion Batteries. *International Conference on E-mobility, Power Control and Smart Systems (ICEMPS),* 1–7. https://doi.org/10.1109/ICEMPS60684.2024.10559331 (2024).

27. Gerbers, R., Mücke, M., Dietrich, F. & Dröder, K. Simplifying Robot Tools by Taking Advantage of Sensor Integration in Human Collaboration Robots. *Procedia CIRP* **44,** 287–292. https://doi.org/10.1016/j.procir.2016.02.135 (2016).

28. Gerbers, R., Wegener, K., Dietrich, F. & Dröder, K. Safe, Flexible and Productive Human-Robot-Collaboration for Disassembly of Lithium-Ion Batteries. in *Recycling of Lithium-Ion Batteries,* edited by A. Kwade & J. Diekmann (Springer International Publishing, 2018), pp. 99–126. https://doi.org/10.1007/978-3-319-70572-9_6.

29. Qu, M. *et al.* Robotic Disassembly Platform for Disassembly of a Plug-In Hybrid Electric Vehicle Battery: A Case Study. *Automation* **5,** 50–67. https://doi.org/10.3390/automation5020005 (2024).

30. Rastegarpanah, A., Ner, R., Stolkin, R. & Marturi, N. Nut Unfastening by Robotic Surface Exploration. *Robotics* **10,** 107. https://doi.org/10.3390/robotics10030107 (2021).

31. Wegener, K., Andrew, S., Raatz, A., Dröder, K. & Herrmann, C. Disassembly of Electric Vehicle Batteries Using the Example of the Audi Q5 Hybrid System. *Procedia CIRP* **23,** 155–160. https://doi.org/10.1016/j.procir.2014.10.098 (2014).

32. Wegener, K., Chen, W. H., Dietrich, F., Dröder, K. & Kara, S. Robot Assisted Disassembly for the Recycling of Electric Vehicle Batteries. *Procedia CIRP* **29,** 716–721. https://doi.org/10.1016/j.procir.2015.02.051 (2015).

33. Zorn, M. *et al.* An Approach for Automated Disassembly of Lithium-Ion Battery Packs and High-Quality Recycling Using Computer Vision, Labeling, and Material Characterization. *Recycling* **7,** 48. https://doi.org/10.3390/recycling7040048 (2022).

34. Mitchell, R. K., Agle, B. R. & Wood, D. J. Toward a Theory of Stakeholder Identification and Salience: Defining the Principle of Who and What Really Counts. *The Academy of Management Review* **22,** 853. https://doi.org/10.2307/259247 (1997).

35. Beghi, M., Braghin, F. & Roveda, L. Enhancing Disassembly Practices for Electric Vehicle Battery Packs: A Narrative Comprehensive Review. *Designs* **7,** 109. https://doi.org/10.3390/designs7050109 (2023).

36. Kampker, A. *et al.* Evaluation Of A Remanufacturing For Lithium Ion Batteries From Electric Cars. *International Journal of Mechanical and Mechatronics Engineering* **10**. https://doi.org/10.5281/zenodo.1128215 (2016).

37. Miloradović, B., Bigorra, E. M., Nolte, T. & Papadopoulos, A. V. Challenges in the Automated Disassembly Process of Electric Vehicle Battery Packs. *IEEE 28th International Conference on Emerging Technologies and Factory Automation (ETFA),* 1–4. https://doi.org/10.1109/ETFA54631.2023.10275389 (2023).

38. Garg, A., Zhou, L., Zheng, J. & Gao, L. Qualitative framework based on intelligent robotics for safe and efficient disassembly of battery modules for recycling purposes. *IOP Conf. Ser.: Earth Environ. Sci.* **463,** 12159. https://doi.org/10.1088/1755-1315/463/1/012159 (2020).

39. Glöser-Chahoud, S. *et al.* Industrial disassembling as a key enabler of circular economy solutions for obsolete electric vehicle battery systems. *Resources, Conservation and Recycling* **174,** 105735. https://doi.org/10.1016/j.resconrec.2021.105735 (2021).

40. Zhu, J. *et al.* End-of-life or second-life options for retired electric vehicle batteries. *Cell Reports Physical Science* **2,** 100537. https://doi.org/10.1016/j.xcrp.2021.100537 (2021).

41. Rosenberg, S., Glöser-Chahoud, S., Huster, S. & Schultmann, F. A dynamic network design model with capacity expansions for EoL traction battery recycling - A case study of an OEM in Germany. *Waste management (New York, N.Y.)* **160,** 12–22. https://doi.org/10.1016/j.wasman.2023.01.029 (2023).

42. Thies, C., Kieckhäfer, K., Hoyer, C. & Spengler, T. S. Economic Assessment of the LithoRec Process. in *Recycling of Lithium-Ion Batteries,* edited by A. Kwade & J. Diekmann (Springer International Publishing, 2018), pp. 253–266. https://doi.org/10.1007/978-3-319-70572-9_15.

43. Lander, L. *et al.* Financial viability of electric vehicle lithium-ion battery recycling. *iScience* **24,** 102787. https://doi.org/10.1016/j.isci.2021.102787 (2021).

44. Lander, L. *et al.* Breaking it down: A techno-economic assessment of the impact of battery pack design on disassembly costs. *Applied Energy* **331,** 120437. https://doi.org/10.1016/j.apenergy.2022.120437 (2023).

45. Ai, Z., Nee, A. Y. C. & Ong, S. K. Artificial Intelligence in Electric Vehicle Battery Disassembly: A Systematic Review. *Automation* **5,** 484–507. https://doi.org/10.3390/automation5040028 (2024).

46. Baazouzi, S., Rist, F. P., Weeber, M. & Birke, K. P. Optimization of Disassembly Strategies for Electric Vehicle Batteries. *Batteries* **7,** 74. https://doi.org/10.3390/batteries7040074 (2021).

47. Yu, H. J., Zhang, T. Z., Yuan, J., Li, C. D. & Li, J. M. Trial Study on EV Battery Recycling Standardization Development. *AMR* **610-613,** 2170–2173. https://doi.org/10.4028/www.scientific.net/AMR.610-613.2170 (2012).

48. Harper, G. D. J. *et al.* Roadmap for a sustainable circular economy in lithium-ion and future battery technologies. *J. Phys. Energy* **5,** 21501. https://doi.org/10.1088/2515-7655/acaa57 (2023).

49. Zhang, J., Li, B., Garg, A. & Liu, Y. A generic framework for recycling of battery module for electric vehicle by combining the mechanical and chemical procedures. *Int J Energy Res* **42,** 3390–3399. https://doi.org/10.1002/er.4077 (2018).

50. Thompson, D. L. *et al.* The importance of design in lithium ion battery recycling – a critical review. *Green Chem.* **22,** 7585–7603. https://doi.org/10.1039/D0GC02745F (2020).

51. Hanisch, C., Diekmann, J., Stieger, A., Haselrieder, W. & Kwade, A. Recycling of Lithium‐Ion Batteries. in *Handbook of Clean Energy Systems,* edited by J. Yan (Wiley, 2015), pp. 1–24. https://doi.org/10.1002/9781118991978.hces221.

52. Windisch-Kern, S. *et al.* Recycling chains for lithium-ion batteries: A critical examination of current challenges, opportunities and process dependencies. *Waste management (New York, N.Y.)* **138,** 125–139. https://doi.org/10.1016/j.wasman.2021.11.038 (2022).

53. Xiao, J., Jiang, C. & Wang, B. A Review on Dynamic Recycling of Electric Vehicle Battery: Disassembly and Echelon Utilization. *Batteries* **9,** 57. https://doi.org/10.3390/batteries9010057 (2023).

54. Mannuß, O. Risks and Mitigation Actions in Automated Disassembly of Traction Batteries. *Proceedings of the 10th Annual World Conference of the Society for Industrial and Systems Engineering* (2021).
